# Agonist-specific conformational dynamics at the β_2-_adrenoceptor dictate allosteric modulation of Gαs signalling and bronchodilation

**DOI:** 10.1111/bph.70513

**Published:** 2026-06-07

**Authors:** Sushrut D. Shah, Joshua Richard, Suvankar Ghosh, Jillian Jerwick, Joice T. Gavali, Raymond B. Penn, Alexander D. MacKerell, Deepak A. Deshpande

**Affiliations:** 1 Center for Translational Medicine, Jane and Leonard Korman Respiratory Institute, Thomas Jefferson University, Philadelphia, Pennsylvania, USA; 2 Department of Pharmaceutical Sciences, School of Pharmacy, University of Maryland Computer-Aided Drug Design Center, University of Maryland, Baltimore, Maryland, USA

**Keywords:** adrenergic receptor, allosteric modulator, asthma, biased agonism, G protein-coupled receptor, pharmacology, β-agonist

## Abstract

**Background and Purpose:**

β_2_-adrenoceptor (β_2_AR) agonists are the cornerstone of asthma therapy and promote bronchodilation through Gαs signalling in airway smooth muscle (ASM), but their efficacy is limited by β-arrestin-mediated β_2_AR desensitization. We recently described allosteric modulators (AMs) of β_2_AR that preferentially enhance isoproterenol-mediated β_2_AR-Gαs signalling in ASM. Orthosteric β-agonists differ in efficacy, kinetics and signalling bias, and how this diversity shapes allosteric modulation of β_2_ARs remains unclear. We investigated whether AMs could selectively modulate β_2_AR-Gαs signalling and bronchodilation in response to endogenous and clinically relevant β-agonists.

**Experimental Approach:**

The effects of a positive AM (PAM37) and two negative AMs (NAM36 and NAM42) were examined on β-agonist-induced Gαs and β-arrestin recruitment and downstream signalling (cAMP, PKA and ERK) in HEK293 cells expressing β_2_ARs and primary human ASM cells. Bronchodilation was assessed using murine precision-cut lung slices and in vivo lung mechanics. Molecular dynamics (MD) simulations were performed to delineate structural determinants of agonist-dependent AM activity.

**Key Results:**

PAM37 enhanced, whereas NAMs attenuated β_2_AR-Gαs coupling, signalling and bronchodilation with no effect on β-arrestin-ERK signalling, establishing Gαs signalling and functional bias. AM effects differed in magnitude and kinetic profiles across β-agonists, with fenoterol uniquely resistant to modulation. MD simulations revealed that fenoterol stabilizes a quasi-inactive β_2_AR conformation characterized by an TM6 shift that occludes the allosteric pocket, providing a structural basis for its resistance.

**Conclusions and Implications:**

These findings establish orthosteric ligand-dependent allostery as a fundamental feature of β_2_AR pharmacology. PAM37 represents a tool to selectively enhance bronchodilation by β-agonists in asthma.

## 1 | INTRODUCTION

Obstructive airway diseases, such as asthma and chronic obstructive pulmonary disease (COPD), affect millions of people globally and represent a major public health burden (Boers et al., 2023; Dharmage et al., 2019; Syamlal et al., 2022). A hallmark feature of these diseases is bronchoconstriction, caused by excessive contraction of airway smooth muscle (ASM), which leads to airflow limitation and breathing difficulty. ASM contraction and relaxation are regulated by various G protein-coupled receptors (GPCRs) (Deshpande & Penn, 2006). Among these, the activation of β_2_-adrenoceptors (β2ARs) promotes airway relaxation and bronchodilation, making β_2_AR agonists (β-agonists) the cornerstone of pharmacological treatment to prevent or reverse bronchoconstriction in asthmatics (Asthma GIf, 2023; Billington et al., 2017). However, the effectiveness of β-agonists is limited by agonist-induced β_2_AR desensitization and internalization with chronic use, and side effects such as increased airway inflammation and mucus production associated with the long-acting β-agonists (Nayak et al., 2019; Salpeter, 2010; Salpeter et al., 2006; Sears, 2013). These limitations underscore the need for developing β_2_AR-targeted therapies that enhance bronchodilation and mitigate the adverse effects of β-agonists.

Endogenously, ASM β_2_ARs are activated by catecholamines, adrenaline and noradrenaline, which contribute to the physiological regulation of airway tone and breathing. Synthetic β_2_AR-selective agonists such as formoterol, albuterol, salmeterol, fenoterol and terbutaline were developed for clinical use. These β-agonists differ in their binding affinity, potency, intrinsic efficacy and duration of action and are classified as partial or full agonists as well as short- or long-acting β-agonists (Barnes, 2006; Billington et al., 2017; Chu & Drazen, 2005; Deshpande & Penn, 2006; Mutlu & Factor, 2008; Pera & Penn, 2016; Shah & Deshpande, 2025). Upon activation by β-agonists, the β_2_AR activates adenylyl cyclase (AC) via GTP-bound active Gαs. Activated AC hydrolyses ATP into cyclic AMP (cAMP), which activates protein kinase A (PKA) that phosphorylates downstream targets and drives ASM relaxation and bronchodilation. However, β-agonist activation also recruits G protein-coupled receptor kinases (GRKs), which phosphorylate agonist-occupied β_2_AR and promote the binding of β-arrestins to the phosphorylated receptor. The β-arrestin-β_2_AR interaction leads to receptor desensitization and subsequent internalization of the receptor via clathrin-coated vesicles (Goodman et al., 1996). In addition to dampening Gαs signalling, β-arrestins initiate alternative signalling pathways, such as the extracellular signal-regulated kinase (ERK) and mitogen-activated protein kinase (MAPK), which contributes to mucus production, ASM proliferation and airway inflammation (Billington & Penn, 2003; Deshpande & Penn, 2022; Gu et al., 2015; Knight et al., 2015; Nguyen et al., 2009; Shah & Deshpande, 2025; Walker et al., 2003). Thus, while β_2_AR-Gαs-cAMP-PKA signalling is therapeutic, β-arrestin-mediated signalling may be detrimental in the context of airway disease. Therefore, selective enhancement of Gαs-cAMP-PKA signalling while limiting the harmful β-arrestin-dependent effects may improve the therapeutic efficacy of β-agonists in asthma.

In our recent study, we discovered a novel class of Gαs-biased allosteric modulators (AMs) of β_2_AR. Using computational modelling, we identified an allosteric binding pocket in an intermediate or near-active receptor conformation of the β_2_AR. Molecular docking studies identified small molecules that bind to this site and modulate canonical β_2_AR-mediated Gαs signalling (Shah et al., 2022). Signalling studies demonstrated that a positive allosteric modulator (PAM), PAM37, enhanced **isoproterenol**-induced cAMP generation and PKA activation, while negative allosteric modulators (NAMs), NAM36 and NAM42, attenuated this response. Importantly, the AMs did not alter β-arrestin recruitment or downstream β-arrestin-mediated ERK activation, indicating selective modulation of the Gαs-cAMP signalling axis. Functional studies using human ASM cells and precision-cut lung slices (PCLS) demonstrated that PAM37 potentiates isoproterenol-induced ASM relaxation and bronchodilation (Shah et al., 2022). While these findings identified a class of β_2_AR AMs that selectively modulate Gαs-cAMP signalling and bronchodilation without affecting β-arrestin pathways, whether these Gαs-biased AMs retain efficacy across a range of endogenous and clinically relevant β-agonists that are known to stabilize distinct β_2_AR conformations remains unknown.

Here, we investigated how orthosteric ligand diversity influences allosteric modulation of β_2_AR signalling and function. We hypothesized that Gαs-biased AMs of β_2_AR differentially modulate Gαs-cAMP signalling and bronchodilation in response to various β-agonists based on the evidence that distinct β-agonists stabilize unique β_2_AR conformation states (Dror et al., 2011a; Liang et al., 2017; Yang et al., 2021; Zhang et al., 2020). To test this, we evaluated the signalling and functional effects of two endogenous catecholamines (adrenaline, noradrenaline) and five synthetic β-agonists (formoterol, albuterol, salmeterol, terbutaline and fenoterol) in the presence of AMs. We assessed β_2_AR-mediated Gαs (cAMP production, PKA activation) and β-arrestin signalling (β-arrestin recruitment and ERK activation) in HEK293 cells expressing human β_2_AR and in primary human ASM cells. Gαs-β_2_AR interactions were further examined using a bioluminescence resonance energy transfer (BRET) assay to determine how AMs regulate the magnitude and kinetics of Gαs coupling. The regulation of ASM function by AMs was assessed ex vivo in murine PCLS and in vivo in murine lung function analyses. Our findings suggest that the AMs bias Gαs-cAMP-PKA signalling across multiple endogenous and clinically relevant β-agonists, augmenting bronchodilation without affecting β-arrestin recruitment. While AMs modulated Gαs signalling and bronchodilation in response to different agonists to varying degrees, they did not alter the signalling and function induced by fenoterol. Molecular dynamics (MD) simulations revealed that fenoterol stabilizes β_2_AR conformations distinct from those induced by isoproterenol, leading to an altered geometry of the allosteric pocket that is structurally less accessible for binding of the AMs. Collectively, these findings define how orthosteric agonist-dependent β_2_AR conformations dictate the functional outcomes of β_2_AR allosteric modulation. Additionally, our studies highlight the potential of Gαs-biased AMs to enhance β_2_AR-targeted therapies in obstructive lung diseases.

## 2 | METHODS

### 2.1 | Materials

The AM compounds were purchased from Chembridge (San Diego, CA, USA) in quantities of 50 mg and dissolved in 10% DMSO to obtain 10-mM stock solutions (Shah et al., 2022). β-agonists (isoproterenol, formoterol, albuterol, salmeterol, adrenaline, noradrenaline and terbutaline) were purchased from Sigma Aldrich (St. Louis, MO, USA) and dissolved in 10% DMSO to prepare a 10-mM stock solution.

### 2.2 | Cell culture

HEK293 cells (RRID:CVCL_0045) were obtained from the American Type Culture Collection (ATCC). Primary human airway smooth muscle (HASM) cells were isolated from donor lungs procured from the National Disease Research Institute as described previously (Panettieri et al., 1989). HEK293 cells were cultured in Dulbecco’s modified Eagle’s medium (DMEM) supplemented with 10% fetal bovine serum (FBS) and penicillin/streptomycin. HASM cells were maintained in Ham’s F-12 medium supplemented with 10% FBS, penicillin and streptomycin, HEPES buffer, CaCl_2_, L-Glutamine and NaOH (Gibco, Waltham, MA, USA), as described previously (Naik et al., 2005; Panettieri et al., 1989; Shah et al., 2022). The use of deidentified human lung tissues was classified as ‘Not Human Subjects Research’ by Thomas Jefferson University’s Institutional Review Board (IRB).

### 2.3 | cDNA constructs and transfections

3xHA-human β_2_AR cloned in a pcDNA3.1 vector was obtained from cDNA Resource Center (www.cdna.org). HEK293 cells were transfected with cDNA encoding human β_2_AR using Lipofectamine 3000 (Invitrogen, Waltham, MA, USA), as described previously (Shah et al., 2022). Cells transiently expressing β_2_ARs were used for experiments or cultured with G418 (Sigma Aldrich) to generate cell lines stably expressing human β_2_ARs. Expression of β_2_ARs was confirmed by western blotting and confocal microscopy using anti-HA antibodies (Naik et al., 2005; Shah et al., 2022).

### 2.4 | cAMP assay

cAMP levels were measured using a commercial ELISA kit (Life Technologies, Waltham, MA, USA), as described previously (Michael et al., 2019; Shah et al., 2022; Yadav et al., 2021). Briefly, HEK293 or HASM cells were grown to full confluence in 24-well plates and serum-starved for 24 h. Cells were incubated with 1-mM IBMX for 10 min before to co-treatment with vehicle or β-agonists (1 nM to 100 μM) and DMSO or β_2_AR AMs for 10 min. Cell lysates were prepared, and cAMP levels were determined as per the manufacturer’s instructions.

### 2.5 | Bioluminescence resonance energy transfer (BRET) assay for Gαs and β-Arrestin2 recruitment to β_2_AR

Recruitment of Gαs and β-arrestin2 to β_2_ARs by β-agonists was evaluated using BRET assays, as described previously (Quoyer et al., 2013; Shah et al., 2022). HEK293 cells in suspension were co-transfected with plasmids encoding β_2_AR-RlucII and either NES-Venus-miniGαs (mGs; a gift from Dr. Nevin Lambert) or β-arrestin2-GFP10 using Metafectene PRO (Biontex, Munich, Germany) and plated onto 96-well white opaque plates. After 48 h, cells were washed with PBS, incubated with 1-μM Coelenterazine 400a (NanoLight Technology, Pinetop, AZ, USA) or Coelenterazine H (Cayman Chemical, Ann Arbor, MI, USA) and co-treated with increasing concentrations of β-agonists in the presence of vehicle or 100-nM AMs. BRET signals were recorded for 20 min using a Tecan Infinite F500 plate reader (RRID:SCR_024561). BRET ratios were calculated by dividing acceptor emission by total donor emission.

### 2.6 | Immunoblotting

HASM cells were grown to near-confluence, serum-starved for 24 h and treated with β-agonists alone or co-treated with 100-nM AMs. For VASP phosphorylation, cells were stimulated with 10-nM isoproterenol, 3.33-nM formoterol, 100-nM albuterol, 100-nM salmeterol, 1-μM adrenaline, 1-μM noradrenaline, 10-nM terbutaline or 100-nM fenoterol to elicit submaximal VASP phosphorylation. Similarly, cells were treated with 100-nM isoproterenol, 100-nM formoterol, 100-nM albuterol, 100-nM salmeterol, 100-nM terbutaline, 100-nM adrenaline, 1-μM noradrenaline or 1-μM fenoterol for ERK phosphorylation. Across these experiments, cells treated with 1-μM forskolin and/or 1-μM isoproterenol served as positive controls. Cells were lysed using RIPA buffer (Cell Signaling Technology, Danvers, MA, USA) supplemented with protease and phosphatase inhibitors (Bimake, Houston, TX, USA). Lysates were separated via SDS-PAGE, proteins transferred to nitrocellulose membranes, and probed with antibodies against VASP (mouse monoclonal; RRID:AB_397822; BD Biosciences, San Jose, CA, USA), phospho-ERK (rabbit monoclonal; RRID:AB_2315112; Cell Signaling Technology) and β-actin (mouse monoclonal; RRID:AB_476744; Sigma-Aldrich, St. Louis, MO, USA). Following incubation with secondary antibodies (RRID:AB_10956588 and RRID:AB_10956166; Li-Cor, Lincoln, NE), signals were detected using the Odyssey scanner (RRID:SCR_022510) and quantified using Image Studio software (RRID:SCR_013715) as per (Naik et al., 2005; Nayak et al., 2019). The immuno-related procedures used comply with the recommendations made by the *British Journal of Pharmacology* (Alexander et al., 2018).

### 2.7 | Radioligand binding assay

HEK293 cells expressing human β_2_ARs were lysed on ice in 50-mM Tris–HCl (pH 7.7) for 10 min, and the membrane was pelleted by centrifugation at 300 × *g* for 5 min. Next, the pellet was washed with 50-mM Tris–HCl (pH 7.7) and pulverized with a 22-gauge needle. Following centrifugation at 27,000 × *g* for 10 min, the pellet was resuspended in binding buffer (15-mM Tris–HCl pH 7.4, 120-mM NaCl, 5.4-mM KCl, 1.8-mM CaCl_2_ and 5-mM glucose). Total membrane protein concentration was determined using a Pierce Bradford assay (ThermoFisher). For the binding assay, 30 μg of membrane protein was incubated with 3 nM [^3^H]-dihydroalprenolol ([^3^H]DHA; PerkinElmer, Waltham, MA, USA) and increasing concentrations of fenoterol (1 nM–100 μM) in the presence of vehicle or 100-nM AMs in a final volume of 500 μl. Non-specific binding was determined using 10-μM alprenolol. Reactions were incubated at 25°C for 2 h and then filtered over 25-mm Whatman GF/B glass microfiber filters (GE Healthcare, Chicago, IL, USA). Filters were washed three times with ice-cold Tris-buffered saline. Radioactivity bound to glass microfiber filters was measured using a TriCarb 4910 TR liquid scintillation analyser (PerkinElmer) and expressed as the percentage of the maximum [^3^H]-DHA-specific binding. Binding competition curves were generated using a nonlinear regression curve-fitting function in GraphPad Prism.

### 2.8 | Ex vivo airway functional studies using murine precision-cut lung slices (mPCLS)

Animals were killed employing CO_2_, followed by exsanguination via severing of the inferior vena cava and hepatic portal vein. Lung slices were prepared from 8- to 12-week-old wild-type BALB/c mice (RRID:IMSR_JAX:000651). Lungs were inflated via tracheal cannulation with 2% low-melting agarose, solidified at 4°C and sectioned (~200 μm thickness) using an oscillating tissue slicer (OTS-5000, FHC Inc.). Slices were cultured for 48 h in DMEM medium supplemented with antibiotic-antimycotic (ThermoFisher Scientific, Waltham, MA, USA) and then pre-contracted with methacholine (MCh, 1 μM) for 10 min to induce bronchoconstriction. Subsequently, slices were treated with increasing concentrations (1 nM–100 μM) of β-agonists in the presence of vehicle or 100-nM AMs. Airways were imaged using an EVOS M7000 Auto microscope (RRID:SCR_025070; Life Technologies), and luminal areas were quantified using the ImageJ (NIH) program. Results were expressed as the percentage change in airway lumen area, normalized to the area at MCh treatment. All animals are housed under a 12-h light–dark cycle, and all animal procedures were approved by the Institutional Animal Care and Use Committee (IACUC) of Thomas Jefferson University. Animal studies are reported in compliance with the ARRIVE guidelines (Percie du Sert et al., 2020) and with the recommendations made by the *British Journal of Pharmacology* (Lilley et al., 2020).

### 2.9 | In vivo airway mechanics

Airway resistance was measured in 8- to 12-week-old BALB/c mice (RRID:IMSR_JAX:000651) using the flexiVent system (RRID:SCR_022673; SCIREQ, Montreal, Canada) as described previously (Deshpande et al., 2010; Shah et al., 2023). Mice were anaesthetised with tribromoethanol (Avertin), intubated and mechanically ventilated. Following baseline recordings, mice were challenged with increasing doses of aerosolized methacholine (1–50 mg·ml^−1^), and changes in lung resistance were recorded. This was followed by co-treatment with aerosolized isoproterenol (0.033–1 mg·ml^−1^), formoterol (0.1–1 mg·ml^−1^), albuterol (0.33–3.33 mg·ml^−1^) or salmeterol (0.1–3.33 mg·ml^−1^) with vehicle or 100-nM AMs. Airway resistance (Rn) was recorded using flexiWare software. Bronchodilation was calculated as the percentage decrease in airway resistance relative to the methacholine response.

### 2.10 | Kinetic feature extraction, clustering and PCA

BRET biosensor tracing for each β-agonist ± 100-nM AMs was normalized to β-agonist (max) and averaged across replicates. To capture both magnitude and temporal dynamics of Gαs recruitment to the β_2_AR, key kinetic features were extracted, including peak, area under the curve (AUC), activation rate (linear fit from baseline to peak), decay slope (linear fit from peak to final time point) and skewness (a measure of asymmetry of the signal), to capture both magnitude and temporal dynamics of Gαs recruitment to the β_2_AR. EC_50_ and E_max_ values were estimated by fitting concentration–response curves with a four-parameter logistic model. Feature vectors for each β-agonist ± 100-nM AMs were constructed by averaging across replicates and subsequently including EC_50_ as an additional feature. Feature vectors were standardized (z-score) prior to PCA and Ward’s hierarchical clustering to assess global differences and similarities in β-agonist kinetic profiles and their modulation by AMs. PCA scores were plotted to visualize relationships among β-agonist, and feature loadings were overlaid as vectors to indicate contributions of individual kinetic parameters. All analyses were performed using Python version 3.11.

### 2.11 | Protein-ligand complex generation

The crystal structure of Isoproterenol (5FW) bound to the active state of the β_2_AR (PDB ID: 3SN6) was collected from the RCSB database (Burley et al., 2017; Rasmussen et al., 2011). Missing residues were modelled using Modeller (v9.11), where the structure with the lowest Discrete Optimized Protein Energy (DOPE) was selected for further studies (Fiser & Sali, 2003). Using that structure, the fenoterol (ZVJ)-β_2_AR complex was generated by structurally aligning fenoterol onto isoproterenol (Figure 8) based on atom numbers 1–12 of both ligands.

### 2.12 | Simulation set-up

CHARMM-GUI membrane builder was used to generate the two active β_2_AR-ligand complexes (3SN6:5FW and 3SN6:ZVJ) (Jo et al., 2008). Termini were capped as NH_3_^+^ and COO^−^ and the disulfide bonds between Cys106-Cys191 and Cys184-Cys190 were maintained. The complexes were embedded in a POPC (1-palmitoyl-2-oleoyl-sn-*glycero*-3-phosphocholine) and cholesterol (9:1) lipid bilayer and solvated with 0.15-M NaCl. The standard CHARMM-GUI protocol for protein-lipid complexes was used for minimization and equilibration (Wu et al., 2014). Minimization was performed by 5000 steps of energy minimizations using the steepest descent method with constraints on non-hydrogen atoms and following by equilibration for 125 ps MD relaxation with 1-fs time step. The temperature was set at 303.15 K using the Berendsen thermostat. A subsequent 11.25-ns MD simulation with a 2-fs time step was performed at 1-bar pressure, maintained using the Berendsen pressure coupling method, and all position restraints were gradually turned off during this relaxation phase. Following the relaxation, a 500-ns production simulation was performed using a 2-fs time step. Temperature and pressure were maintained at 303.15 K and 1 bar using the Nosé-Hoover thermostat and Parrinello-Rahman barostat, respectively (Hoover, 1985; Nosé, 1984; Rahman, 1981). A harmonic spring restraint of 20 kJ·mol^−1^·nm^2^ was applied between the ligand (C9 atom, Figure 8) and protein (Cα atoms of residues Asp113^3.32^, Ser204^5.43^, Phe289^6.51^ and Asn312^7.39^) during the initial 100 ns of the simulation to allow relaxation of the protein-ligand environment. The restraint was subsequently removed, and the remaining 400 ns were performed without any restraints. Relaxation and production simulations employed the LINCS algorithm to constrain covalently bonded hydrogens and the Leap-Frog integrator for time integration. Long-range electrostatic interactions were treated using the particle mesh Ewald (PME) method with a real-space cut-off of 12 Å, cubic B-spline interpolation and a maximum grid spacing of 1.2 Å (Darden et al., 1993; Hess et al., 1998). All simulations were performed using OPENMM (version 8.1) with the CHARMM36 force field parameters for proteins and lipids (Eastman et al., 2024; Huang et al., 2017; Yu et al., 2021). Ligand parameters were generated using CGenFF, and water molecules were modelled using the TIP3P water model (Jorgensen et al., 1983; Vanommeslaeghe et al., 2012; Vanommeslaeghe & MacKerell, 2012).

Each of the two complexes was simulated in three independent 500-ns MD simulations. The three trajectories were performed with different initial velocities randomly assigned from a Maxwell–Boltzmann distribution at 303.15 K. Single-snapshot results were obtained from the final time frame of the first simulation, with distributions and average results, along with error analysis, obtained over the final 100 ns of the three simulations. Trajectory coordinates were saved every 10 ps for subsequent analysis.

### 2.13 | Structure analysis

To assess the effect of the orthosteric ligands on the known allosteric pocket, the distance between transmembrane (TM) helices TM3 and TM6 (Figure 8) was monitored by measuring the separation between the Cα atoms of residues Arg131^3.50^ and Glu268^6.30^, which form the ionic lock (Dror et al., 2011b). Distances of four transmembrane helices (TM3-TM5-TM6-TM7; Figure 8), based on Arg131^3.50^ (TM3), Phe223^5.62^ (TM5), Tyr274^6.36^ (TM6) and Arg328^7.55^ (TM7), were also measured to further assess the allosteric pocket shape. The final 100 ns of each trajectory were used for analysis. MDAnalysis and PyMOL were used to analyse the data (Michaud-Agrawal et al., 2011; Yuan et al., 2017).

### 2.14 | Statistical analysis

All data are presented as mean ± SEM. For HASM cells and murine tissue/in vivo studies, ‘*n*’ represents biologically independent samples (different lung donors) or the number of animals. For HEK293 experiments, ‘n’ indicates technical replicates. cAMP concentration was determined by extrapolation from standard curves. EC_50_ and E_max_ values were calculated using non-linear regression (four-parameter logistic fit) analysis using GraphPad Prism v10 (RRID:SCR_002798). Statistical analysis was carried out only where the number of independent experiments, n, was 5 or more. For experiments where n<5, statistical analysis was not carried out and results should be regarded as preliminary. Statistical significance was assessed using one-way or two-way ANOVA followed by Bonferroni post hoc test or unpaired Student’s *t* test, as appropriate. Post-hoc tests were run only if F achieved P<0.05 and there was no significant variance inhomogeneity. A P-value ≤ 0.05 was considered statistically significant. Data and statistical analysis complied with the recommendations of the *British Journal of Pharmacology* on experimental design and analysis in pharmacology (Curtis et al., 2025).

### 2.15 | Nomenclature of targets and ligands

Key protein targets and ligands in this article are hyperlinked to corresponding entries in the IUPHAR/BPS Guide to PHARMACOLOGY http://www.guidetopharmacology.org and are permanently archived in the Concise Guide to PHARMACOLOGY 2025/26 (Alexander, Davenport et al., 2025; Alexander, Fabbro et al., 2025).

## 3 | RESULTS

### 3.1 | Allosteric modulators demonstrate agonist specificity in modulating cAMP generation

Different β-agonists exhibit variability in G protein recruitment, cAMP generation and β-arrestin recruitment to the β_2_AR (Shah & Deshpande, 2025) (De Pascali et al., 2022). Our previous study demonstrated that PAM37 augments, while NAM36 and NAM42 inhibit isoproterenol-stimulated cAMP production (Shah et al., 2022). Here, we evaluated the effects of the AMs PAM37, NAM36 and NAM42 on cAMP generation by a panel of endogenous and clinically relevant β-agonists with disparate pharmacological properties. HEK293 cells expressing human β_2_AR were treated with vehicle or AMs (100 nM) and increasing concentrations of β-agonists. We tested PAM37, NAM36 and NAM42 at concentrations ranging from 0.1 nM to 10 μM in our previous study and determined that 100 nM produced a robust modulation of signalling without reaching full saturation. Therefore, in the studies described here, we used 100 nM of AMs. cAMP was measured by ELISA. All β-agonists increased cAMP in a dose-dependent manner (Figure S1A), although the EC_50_ and E_max_ varied among β-agonists. PAM37 enhanced cAMP production in response to isoproterenol, formoterol, albuterol, salmeterol, adrenaline, terbutaline and noradrenaline (Figure S1C–H; Table S5A). Similarly, NAM36 and NAM42 inhibited cAMP generation by most of the agonists. Specifically, NAM36 and NAM42 inhibited cAMP induced by isoproterenol, formoterol, albuterol, salmeterol, adrenaline and noradrenaline. However, none of the AMs modulated fenoterol-induced cAMP production (Figure S1B–H).

**FIGURE 1:**
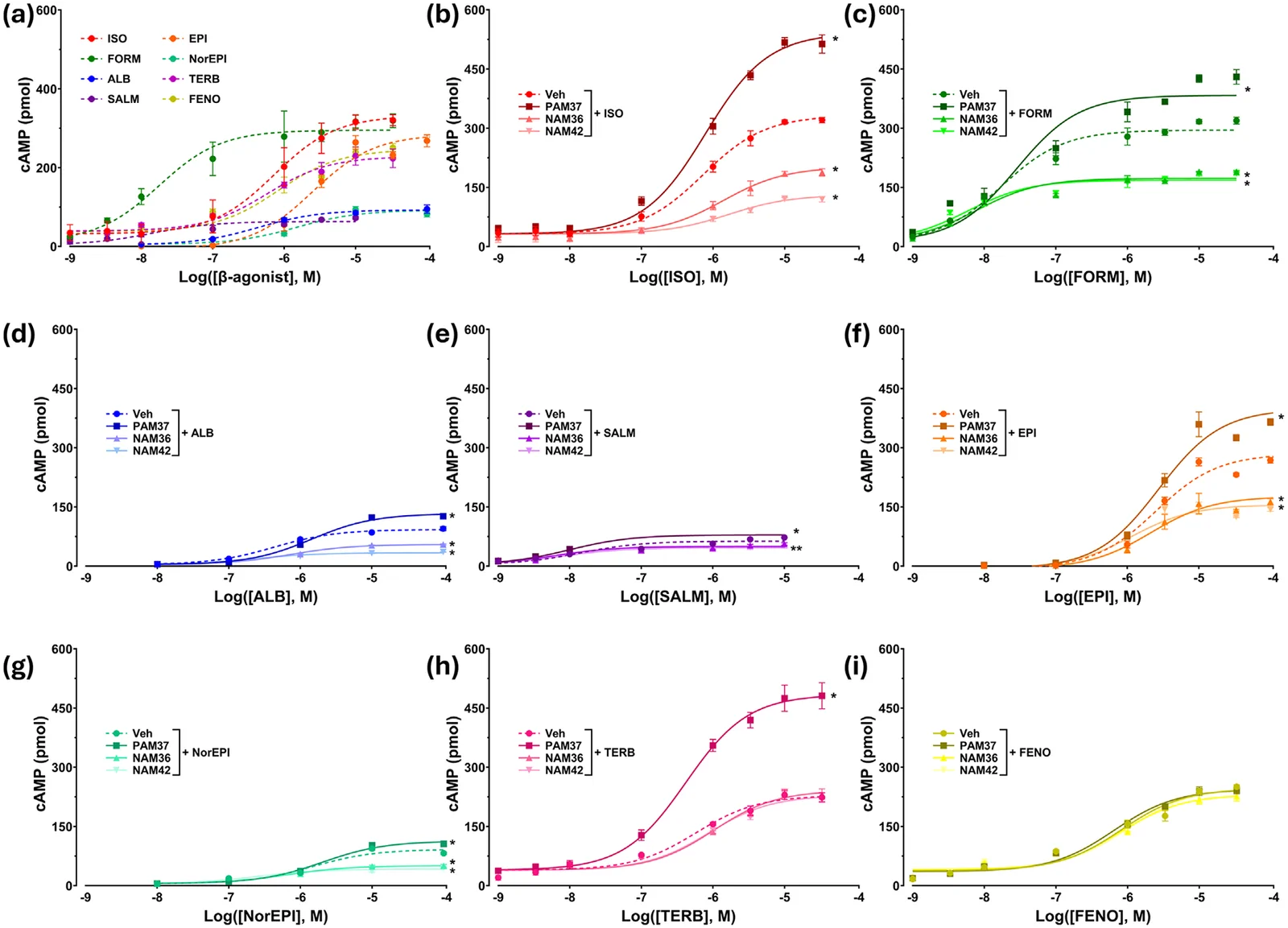
Differential modulation of various β-agonist-induced cAMP generation in primary human ASM cells expressing endogenous β_2_-adrenoceptors by the AMs. Human ASM cells were plated in 24-well plates and stimulated with 100-nM AMs or vehicle in the presence of increasing concentrations of various β-agonists for 10 min. Generation of cAMP was measured by ELISA, and dose–response curves were generated using a non-linear logistic curve from multiple experiments. Dose–response curves of (a) various β-agonists alone or (b–i) in the presence of 100-nM AMs. PAM37 augmented, and NAM36 and NAM42 inhibited cAMP generation induced by (b) ISO (n = 10), (c) FORM (n = 10), (d) ALB (n = 6), (e) SALM (n = 8), (f) EPI (n = 7) and (g) NorEPI (n = 5) but had a variable effect with (h) TERB (PAM augmented and but NAMs did not inhibit; n = 5) and no effect with (i) FENO (n = 4). n = 4–10. Statistical analysis was performed using a two-way ANOVA with a Bonferroni post hoc test. *P < 0.05 β-agonist versus AMs + β-agonist. As n= 4 for experiments with FENO, statistical analysis was not carried out. ALB: albuterol; EPI: adrenaline; FENO: fenoterol; FORM: formoterol; ISO: isoproterenol; NAM: negative allosteric modulator; NorEPI: noradrenaline; PAM: positive allosteric modulator; SALM: salmeterol; TERB: terbutaline.

In primary human airway smooth muscle (HASM) cells (which express endogenous β_2_ARs), the β-agonists displayed heterogeneity in inducing intracellular cAMP levels consistent with the data in HEK293 cells (Figure 1a). PAM37 enhanced and NAM36 and NAM42 inhibited cAMP generation by isoproterenol, formoterol, albuterol, salmeterol, adrenaline, terbutaline and noradrenaline, whereas fenoterol-induced cAMP generation was unaffected (Figure 1b–i). E_max_ and EC_50_ values were calculated for β-agonists-induced cAMP generation in HEK293 and HASM cells (Tables S1A,B and S2A,B). While β-agonist EC_50_ values were minimally affected by AMs, the efficacy (E_max_) was significantly affected. Analysis of maximal responses revealed that PAM37 enhanced and NAM36 and NAM42 inhibited cAMP generation by all agonists except fenoterol. The increase in cAMP production by PAM37 in HASM cells was most pronounced for terbutaline, followed by isoproterenol, adrenaline, formoterol, albuterol, noradrenaline and salmeterol (Table S5A). NAM36 decreased cAMP production by noradrenaline, albuterol, isoproterenol, formoterol and adrenaline, whereas NAM42 decreased production by albuterol, isoproterenol, noradrenaline, adrenaline, formoterol and salmeterol (Table S5A). Similar to HASM cells, the magnitude of modulation of cAMP generation by the AMs was agonist-dependent in HEK293 cells, with the highest degree of modulation by PAM37 in isoproterenol, followed by terbutaline, salmeterol, adrenaline, formoterol, albuterol and noradrenaline (Table S5B). NAM36 decreased cAMP production by isoproterenol, followed by albuterol, formoterol, noradrenaline, adrenaline and salmeterol. NAM42 exhibited the greatest decrease in cAMP generation in response to isoproterenol, adrenaline, albuterol, formoterol, noradrenaline, salmeterol and terbutaline (Table S5B). Notably, NAM36 and NAM42 did not affect maximal responses to fenoterol. Collectively, these findings establish orthosteric ligand-dependency as a key factor in the modulation of β_2_AR signalling by the AMs.

### 3.2 | β-agonist-induced vasodilator-stimulated phosphoprotein (VASP) phosphorylation is augmented by PAM37

**FIGURE 2:**
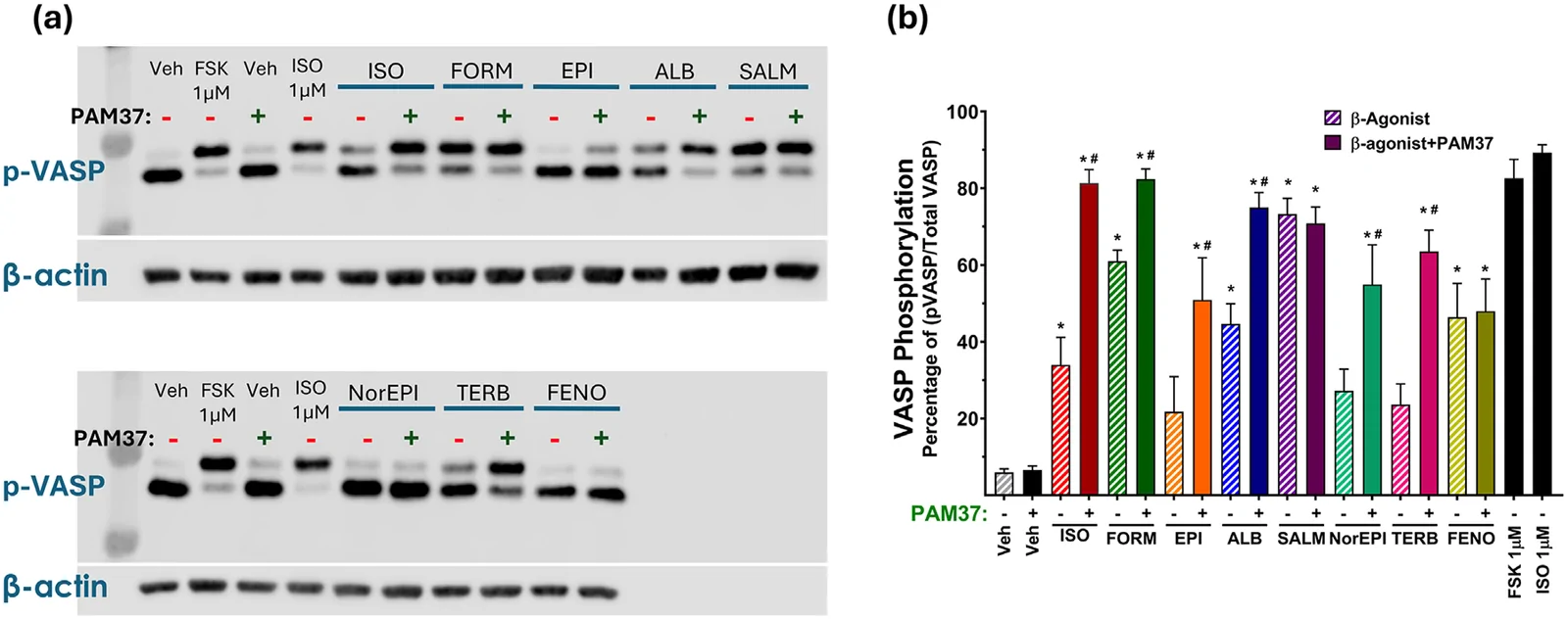
Effect of PAM37 on PKA signalling induced by various β-agonists. To examine the downstream signalling, human ASM cells were plated in 24-well plates and stimulated with 100-nM PAM37/vehicle in the presence of various β-agonists for 10 min. β-agonists were used at the following concentrations: 10-nM isoproterenol (ISO), 3.33-nM formoterol (FORM), 100-nM albuterol (ALB), 100-nM salmeterol (SALM), 1-μM adrenaline (EPI), 1-μM noradrenaline (NorEPI), 10-nM terbutaline (TERB) and 100-nM fenoterol (FENO). PKA phosphorylation was measured by western blotting to examine a downstream substrate, VASP. (a) Representative western blot of various β-agonists in the presence of vehicle or 100-nM PAM37 or positive controls, forskolin and ISO. (b) Quantified band intensities of phosphorylated VASP (top band) normalized to total VASP (both bands). As expected, ISO and adenylyl cyclase activator forskolin induced VASP phosphorylation. PAM37 augmented VASP phosphorylation induced by ISO (n = 8), FORM (n = 8), ALB (n = 8), EPI (n = 6), NorEPI (n = 6) and TERB (n = 8), but not by SALM (n = 8) and FENO (n = 6). n = 6–8. Statistical analysis was performed using a two-way ANOVA with a Bonferroni post hoc test. *P < 0.05 vehicle versus β-agonist ± PAM37, ^#^P < 0.05 β-agonist versus AMs + β-agonist. ALB: albuterol; EPI: adrenaline; FENO: fenoterol; FORM: formoterol; ISO: isoproterenol; NorEPI: noradrenaline; PAM: positive allosteric modulator; SALM: salmeterol; TERB: terbutaline.

cAMP activates PKA, which in turn phosphorylates various cellular target proteins, one of which is VASP, which we have determined to be a sensitive indicator of PKA activity in ASM cells (Guo et al., 2005). Primary HASM cells were treated with various β-agonists in the presence of vehicle or 100-nM PAM37. β-Agonists (isoproterenol, formoterol, adrenaline, albuterol, salmeterol, noradrenaline, fenoterol and terbutaline) induced phosphorylation of VASP to varying levels. PAM37 further augmented VASP phosphorylation in response to treatment with isoproterenol, formoterol, albuterol, adrenaline, noradrenaline and terbutaline (Figure 2). However, PAM37 did not modulate VASP phosphorylation in fenoterol-treated cells (Figure 2), consistent with the lack of effect on cAMP generation described above.

### 3.3 | Allosteric modulators do not modulate β-agonist-mediated β-arrestin recruitment to the β_2_-adrenoceptor or ERK activation

**FIGURE 3:**
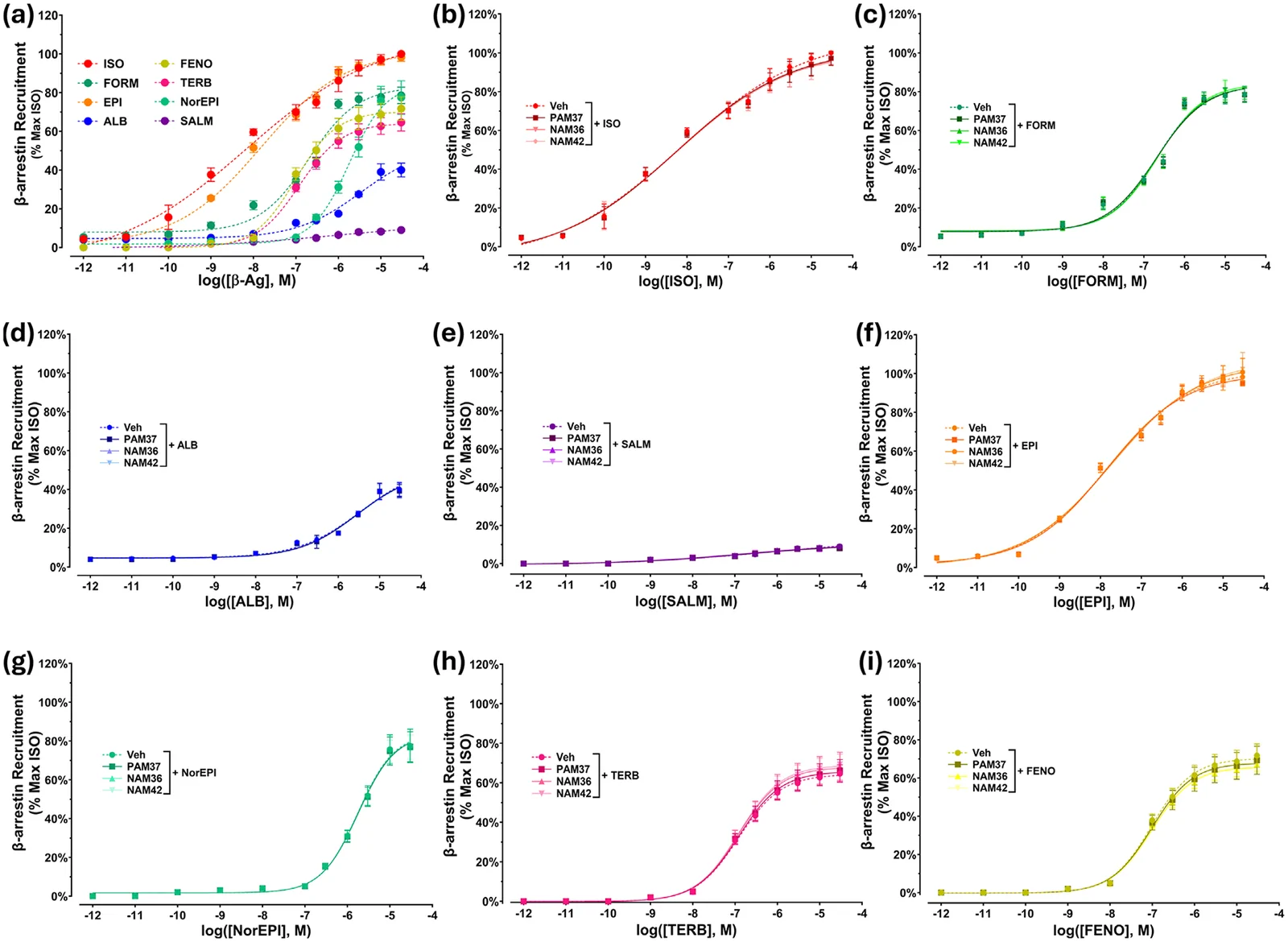
Allosteric modulators do not modulate β-agonist-induced β-arrestin recruitment to the β_2_AR. HEK293 cells expressing β_2_AR-RlucII and β-arrestin2-GFP BRET reporters were stimulated with 100-nM AMs/vehicle in the presence of increasing concentrations of various β-agonists. (a–i) β-agonists induced β-arrestin2 recruitment to β_2_AR in a dose-dependent manner. While various β-agonists had disparate levels of β-arrestin recruitment to β_2_ARs, AMs failed to modulate this further for all β-agonists: (b) ISO (n = 5), (c) FORM (n = 5), (d) ALB (n = 5), (e) SALM (n = 5), (f) EPI (n = 5), (g) NorEPI (n = 5), (h) TERB (n = 5) and (i) FENO (n = 4). n = 4–5. ALB: albuterol; EPI: adrenaline; FENO: fenoterol; FORM: formoterol; ISO: isoproterenol; NorEPI: noradrenaline; PAM: positive allosteric modulator; SALM: salmeterol; TERB: terbutaline.

GPCR kinases (GRKs) phosphorylate β-agonist-occupied β_2_ARs, promoting β-arrestin binding, which leads to arrestin-mediated ERK activation and receptor internalization and desensitization. To investigate the effect of AMs on β-arrestin recruitment to β_2_AR and ERK activation by different β-agonists, we used a BRET assay and immunoblot analysis, respectively. HEK293 cells were transfected with the BRET biosensors β_2_AR-RlucII and β-arrestin-GFP10 and stimulated with various β-agonists at increasing concentrations in the presence of vehicle or 100-nM AMs. All the β-agonists promoted β-arrestin recruitment to β_2_ARs in a dose-dependent manner (Figure 3a). Neither PAM37 nor NAM36 and NAM42 modulated β-agonist-mediated increases in β-arrestin recruitment to the β_2_AR (Figure 3b–i). EC_50_ and E_max_ values are summarized in Table S3. Moreover, PAM37, NAM36 and NAM42 did not modulate β-agonist-induced ERK phosphorylation in HASM cells (Figure S2). Collectively, these studies suggest that AMs selectively modulate Gαs-cAMP but not β-arrestin pathways activated by multiple endogenous and clinically relevant β-agonists.

### 3.4 | PAM37 augments and NAM36 and NAM42 inhibit β-agonist-mediated bronchodilation

**FIGURE 4:**
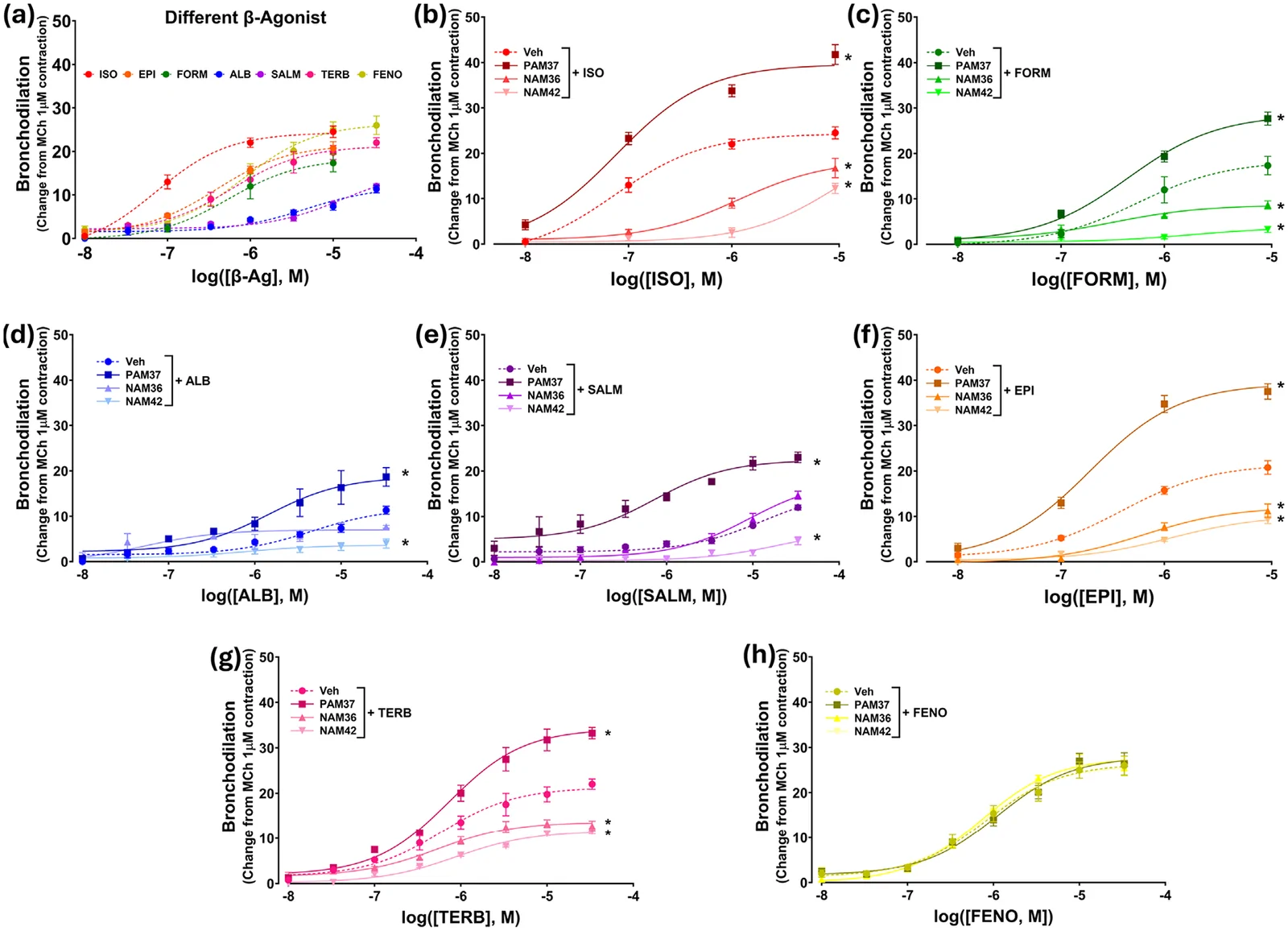
β_2_-Adrenoceptor AMs differentially modulate airway relaxation induced by different β-agonists. Mouse precision-cut lung slices (PCLS) were employed to examine the effect of AMs on the airways by generating 200-μm-thick murine lung slices. PCLS were contracted with 1-μM methacholine to simulate bronchoconstriction and then stimulated with 100-nM AMs/vehicle in the presence of increasing concentrations of β-agonists to induce bronchodilation. Changes in airway lumen area were quantified using ImageJ to determine the magnitude of bronchodilation. Dose–response curves from multiple experiments were generated using a non-linear logistic curve. Dose–response curves of (a) various β-agonists alone or (b–h) in the presence of 100-nM AMs. PAM37 augmented and NAM36 and NAM42 inhibited bronchodilation induced by (b) ISO (n = 10), (c) FORM (n = 6), (d) ALB (n = 6), (e) SALM (n = 6), (f) EPI (n = 6) and (g) TERB (n = 6) but had no effect on (h) FENO (n = 4) in isolated murine slices (n=4). n = 4–10. Statistical analysis was performed using a two-way ANOVA with a Bonferroni post hoc test. *P < 0.05 β-agonist versus AMs + β-agonist. As n= 4 for experiments with FENO, statistical analysis was not carried out. ALB: albuterol; EPI: adrenaline; FENO: fenoterol; FORM: formoterol; ISO: isoproterenol (n=4)l; NAM: negative allosteric modulator; NorEPI: noradrenaline; PAM: positive allosteric modulator; SALM: salmeterol; TERB: terbutaline.

β-Agonists activate the cAMP-PKA pathway and elicit bronchodilation in the airways. Ex vivo murine precision-cut lung slices (mPCLS) were used to assess the effect of AMs on bronchodilation by different β-agonists. Treatment of mPCLS with different β-agonists elicited varying magnitudes of bronchodilation in the airways in a concentration-dependent manner (Figure 4a), consistent with previous studies. The efficacy of β-agonists in bronchodilating or relaxing ASM tends to correlate with agonist intrinsic activity (Nials et al., 1993; Shah & Deshpande, 2025). PAM37 significantly enhanced bronchodilation by isoproterenol, formoterol, albuterol, salmeterol, adrenaline and terbutaline (Figure 4b–g). NAM36 and NAM42 inhibited β-agonist-mediated bronchodilation, with differences in the degree of inhibition between NAM36 and NAM42 for specific β-agonists (Figure 4b–g). AMs had no effect on fenoterol-induced bronchodilation, consistent with cAMP/PKA data described above. The E_max_ and EC_50_ values for bronchodilation induced by different β-agonists ± AMs in mPCLS are summarized in Table S4. Notably, while the AMs significantly modulated the maximal efficacy (E_max_) of the β-agonists, they only had a modest effect on their respective potencies (EC_50_) (Table S4). The magnitude of functional allosteric modulation was also agonist dependent (Table S5C). PAM37 most effectively enhanced salmeterol-induced bronchodilation, followed by adrenaline, isoproterenol, albuterol, formoterol and terbutaline. NAM36 most effectively decreased formoterol-induced bronchodilation, followed by adrenaline, terbutaline, isoproterenol and albuterol, whereas NAM42 demonstrated the greatest inhibition of agonist-induced bronchodilation with formoterol, followed by albuterol, salmeterol, adrenaline, isoproterenol and terbutaline (Table S5C). Overall, these findings suggest that PAM37 enhances—and NAM36 and NAM42 inhibit—β-agonist-induced bronchodilation in mPCLS.

### 3.5 | PAM37 enhances β-agonist-mediated bronchodilation in vivo

**FIGURE 5:**
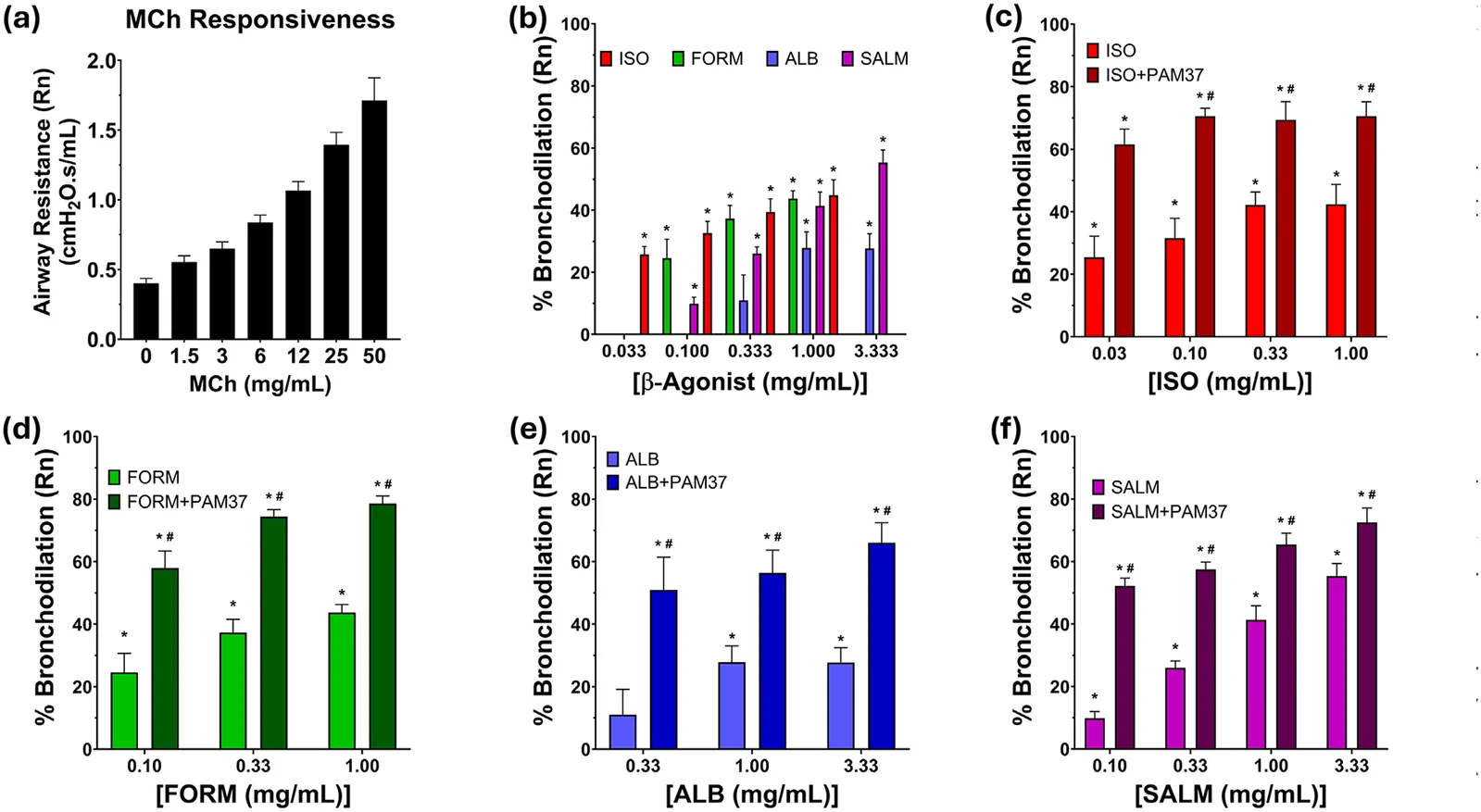
PAM37 augments bronchodilation induced by clinically relevant β-agonists. To examine the in vivo effects of PAM37 on airway mechanics, the trachea in anaesthetised mice was intubated and ventilated using the flexiVent system according to IACUC-approved protocol. Mice were subjected to aerosolized methacholine to induce bronchoconstriction and then treated with β-agonists in the presence of vehicle/100-nM PAM37. (a) Increasing doses of methacholine increased airway resistance. (b) Different β-agonists relaxed the methacholine-induced bronchodilation. PAM37 augmented bronchodilation induced by (c) ISO (n = 6), (d) FORM (n = 7), (e) ALB (n = 9) and (f) SALM (n = 10). n = 6–10. Statistical analysis was performed using a two-way ANOVA with a Bonferroni post hoc test. *P < 0.05 vehicle versus β-agonist ± PAM37, ^#^P < 0.05 β-agonist versus PAM37 + β-agonist. ALB: albuterol; FORM: formoterol; ISO: isoproterenol; PAM: positive allosteric modulator; SALM: salmeterol.

To evaluate the in vivo efficacy of AMs, we assessed lung functions in mice using the flexiVent (Scireq) system (Deshpande et al., 2010; Shah et al., 2023). We used the clinically relevant β-agonists (formoterol, albuterol and salmeterol) along with the prototypical full agonist, isoproterenol, in these studies. Aerosol treatment of mice with increasing doses of methacholine (MCh) increased airway resistance (Figure 5a). Subsequent administration of β-agonists reduced the MCh-induced increase in airway resistance in a dose-dependent manner (Figure 5b). PAM37 significantly enhanced bronchodilation mediated by isoproterenol, formoterol, albuterol and salmeterol (Figure 5c–f). These findings underscore the therapeutic potential of PAM37 in enhancing the bronchodilatory effects of β-agonists.

### 3.6 | Allosteric modulator modulate Gαs-protein binding to the β_2_-adrenoceptor upon agonist stimulation

**FIGURE 6:**
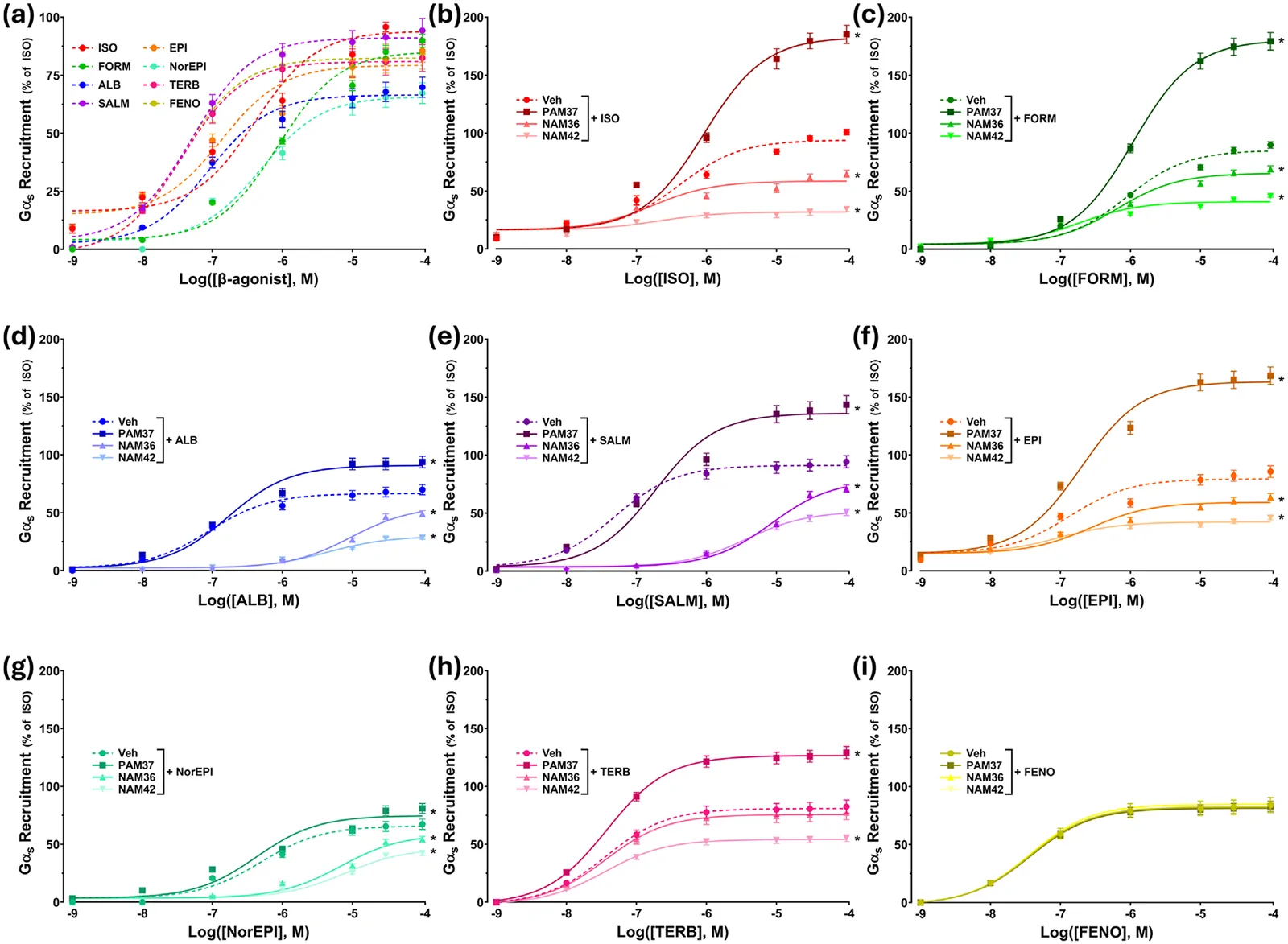
β_2_-Adrenoceptor AMs differentially modulate Gαs-recruitment to the β_2_AR induced by different β-agonists. HEK293 cells expressing β_2_AR-RlucII and Gαs-YFP BRET reporters were stimulated with 100-nM AMs/vehicle in the presence of increasing concentrations of various β-agonists. Dose–response curves from multiple experiments were generated using a non-linear logistic curve. (a) β-agonists elicited a broad range of maximal Gαs recruitment to β_2_AR in a dose and agonist-dependent manner. PAM37 augmented and NAM36 and NAM42 inhibited bronchodilation induced by (b) ISO (n = 12), (c) FORM (n = 8), (d) ALB (n = 8), (e) SALM (n = 12), (f) EPI (n = 12) and (g) NorEPI (n = 9) but had a variable effect with (h) TERB (n = 7; NAM36 did not inhibit) and no effect with (i) FENO (n = 6). n = 6–12. Statistical analysis was performed using a two-way ANOVA with a Bonferroni post hoc test. *P < 0.05 β-agonist versus AMs + β-agonist. ALB: albuterol; EPI: adrenaline; FENO: fenoterol; FORM: formoterol; ISO: isoproterenol; NAM: negative allosteric modulator; NorEPI: noradrenaline; PAM: positive allosteric modulator; SALM: salmeterol; TERB: terbutaline.

β-Agonists binding to β_2_AR result in the coupling of Gαs protein to the β_2_AR (De Pascali et al., 2022). We assessed the effect of AMs on β-agonist-mediated Gαs coupling to β_2_AR using a BRET biosensor expressed in HEK293 cells. Each β-agonists promoted Gαs interaction with the β_2_AR in a dose-dependent manner (Figure 6a). PAM37 augmented, whereas NAM36 and NAM42 inhibited isoproterenol, formoterol, albuterol, salmeterol, adrenaline, noradrenaline and terbutaline-induced Gαs interaction with the β_2_AR (Figure 6b–i). Calculated EC_50_ and E_max_ values are represented in Table 1. PAM37 produced the greatest enhancement with formoterol, followed by adrenaline, isoproterenol, terbutaline, salmeterol, albuterol and noradrenaline (Table S5D). Conversely, the NAMs exhibited distinct rank orders of inhibition: NAM36 most effectively decreased the Gαs interaction induced by isoproterenol, followed by albuterol, adrenaline, salmeterol, formoterol and noradrenaline, whereas NAM42 demonstrated the greatest inhibition with isoproterenol, followed by albuterol, formoterol, adrenaline, salmeterol, noradrenaline and terbutaline. Notably, neither PAM37 nor NAM36 and NAM42 modulated fenoterol-mediated Gαs recruitment to the β_2_AR.

### 3.7 | Kinetic analysis of β-agonist-induced Gαs coupling to the β_2_-adrenoceptor and its modulation by allosteric modulators

To further characterize the dynamics of β_2_AR-Gαs coupling, we quantified the kinetic features from dose–response traces of Gαs-coupling to β_2_AR. BRET traces revealed distinct kinetic signatures among β-agonists (Figure S3), with agonists such as isoproterenol and formoterol exhibiting faster activation rates than salmeterol, albuterol and noradrenaline. Further quantitative analyses confirmed agonist-specific differences in activation rate, area under the curve, peak (max signal) and EC_50_ (Figure S3B–E). These multivariate features were then used in a global analysis, in which principal component analysis (PCA) was applied to capture variance associated with agonist differences. Ward’s hierarchical clustering was used to group agonists based on feature similarity. Together, these analyses generated agonist–feature profiles. Isoproterenol, fenoterol, adrenaline and terbutaline clustered together, characterized by higher area under the curve (AUC), faster activation rate and greater peak responses, consistent with rapidly acting, high-efficacy agonists. In contrast, albuterol, salmeterol and noradrenaline formed a distinct cluster with reduced activation rates and lower overall signalling output, indicating a slower onset and/or reduced efficacy (Figure S3F–H). Taken together, these data demonstrate that β-agonists can be stratified based on their kinetic signalling profiles, which align with their known pharmacological classifications and onset-of-action characteristics. This suggests that early signalling events at the receptor-G protein interface may play an important role in shaping the speed and potency with which these drugs act in physiological and therapeutic settings.

**FIGURE 7:**
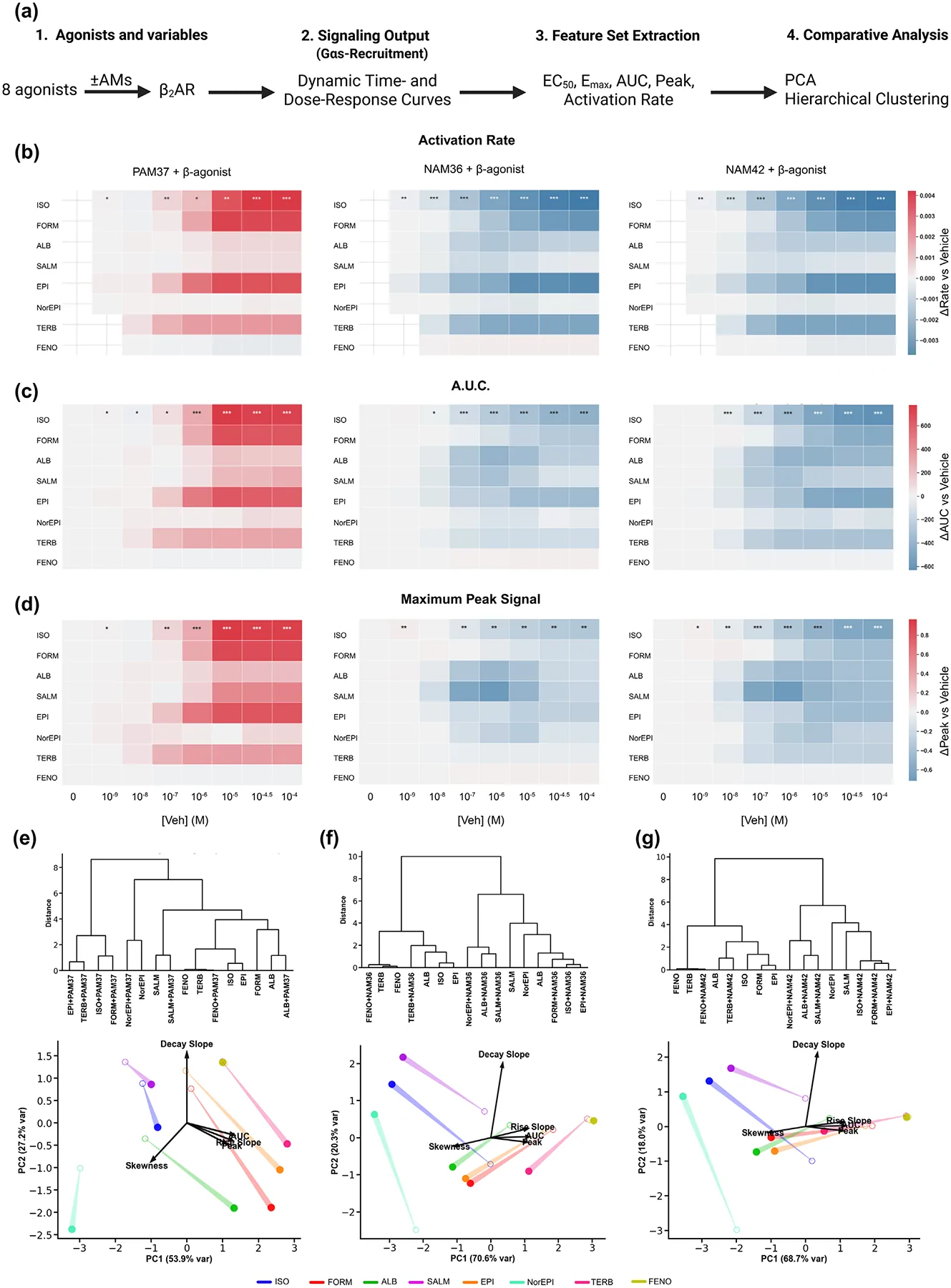
Allosteric modulators reshape the kinetic landscape of β-agonist–induced Gαs coupling to the β_2_AR. (a) Schematic showing workflow for the kinetic analysis of Gαs recruitment. Dose- and time-dependent Gαs recruitment profiles were generated for eight β-agonists (ISO, FORM, ALB, SALM, EPI, NorEPI, TERB and FENO) in the presence or absence of AMs and used to derive key kinetic and pharmacological parameters, including EC_50_, E_max_, AUC, peak response and activation rate. These features were integrated and subjected to principal component analysis (PCA) and hierarchical clustering to define agonist-specific signalling signatures. This approach enabled evaluation of how AMs reshape β-agonist–specific kinetic features. The effects of PAM37 (left panel), NAM36 (middle panel) and NAM42 (right panel) on each of the β-agonists’ profiles of Gαs coupling to β_2_AR. (b–d) Heat maps illustrate the change in (b) activation rate, (c) area under the curve and (d) maximal peak signal across different β-agonists and doses. (e–g) To assess how AMs reshape agonist signalling signatures, hierarchical clustering (top panels) and principal component analysis (PCA, bottom panels) were performed on features derived from Gαs recruitment kinetics in the presence of (e) PAM37, (f) NAM36 and (g) NAM42. In the PCA plots, open circles represent the kinetic signature of the agonist alone, while the corresponding filled circles show the profile of the agonist in the presence of the AM; lines connect the two conditions to visualize the shift. PAM37 shifted the kinetic profiles of most agonists towards a more robust phenotype, while NAM36 and NAM42 shifted them towards a weaker phenotype, with fenoterol remaining unaffected. ALB: albuterol; AUC: area under the curve; EPI: adrenaline; FENO: fenoterol; FORM: formoterol; ISO: isoproterenol; NAM: negative allosteric modulator; NorEPI: noradrenaline; PAM: positive allosteric modulator; SALM: salmeterol; TERB: terbutaline.

We next examined the impact of AMs on the Gαs activation profile of β-agonists (Figures 7a and S4). PAM37 enhanced Gαs coupling, significantly increasing one or more kinetic features for most β-agonists, except for fenoterol, for which no effect was observed. In contrast, NAM36 and NAM42 broadly reduced signalling, except in the case of fenoterol. Among β-agonists, isoproterenol was the most strongly potentiated or attenuated by PAM37 and NAM36 and NAM42, respectively (Figures 7b–d and S5). To evaluate global reshaping of the kinetic landscape, we applied Wards clustering using the derived kinetic features as the component space. This analysis revealed consistent shifts in the kinetic profiles induced by PAM37, with most agonists exhibiting faster activation rates and higher area under the curve (AUC) and peak responses. Notably, noradrenaline showed increased skewness relative to the agonist alone, suggesting a subtle shift towards a faster onset of Gαs coupling (Figure 7e). In contrast, NAM36 and NAM42 tended towards a weaker signalling profile, predominantly driven by reductions in the activation rate, area under the curve (AUC) and the peak (Figure 7f,g).

Having established that AMs consistently shift agonist kinetic profiles at a global level, we next examined how these AMs influence the temporal dynamics and functional potency of Gαs-coupled signalling for each β-agonist. Temporal analyses revealed potentiation or attenuation of Gαs coupling promoted by PAM37 or NAM36 and NAM42, respectively (Figure S4B). Furthermore, we plotted EC_50_ and E_max_ values for Gαs coupling to highlight the differences among AMs in β-agonist-mediated potency and efficacy profiles over time (Figure S4C). The change in efficacy illustrates how PAM37 consistently shifted agonists towards higher efficacy, whereas NAM36 and NAM42 shifted them towards lower signalling output. Notably, AMs exhibited kinetic profiles of potency and efficacy similar to those of full or near full agonists (isoproterenol, formoterol, and adrenaline). Together, these results suggest that AMs not only modulate the magnitude of β-agonist signalling but also reshape the temporal and kinetic landscape of receptor activation.

### 3.8 | Allosteric modulators do not alter the binding affinity of fenoterol to the β_2_-adrenoceptor

One potential reason for the lack of fenoterol-induced signalling modulation by AMs is that AMs alter fenoterol binding affinity for β_2_ARs. To investigate this, we performed competitive radioligand binding assays using [^3^H]-dihydroalprenolol ([^3^H]DHA) in membranes from HEK293 cells stably expressing the human β_2_AR. Fenoterol displaced [^3^H]DHA binding in a concentration-dependent manner, suggesting fenoterol binding to β_2_AR (Figure S6). Notably, co-incubation with AMs (PAM37, NAM36 and NAM42) did not alter the binding affinity or maximal binding of fenoterol to the β_2_AR. These data demonstrate that the inability of AMs to modulate fenoterol-induced Gαs-cAMP-PKA signalling is not due to changes in β_2_AR binding affinity.

### 3.9 | Computational modelling reveals differences in allosteric pocket availability in isoproterenol- and fenoterol-bound β_2_-adrenoceptor

**FIGURE 8:**
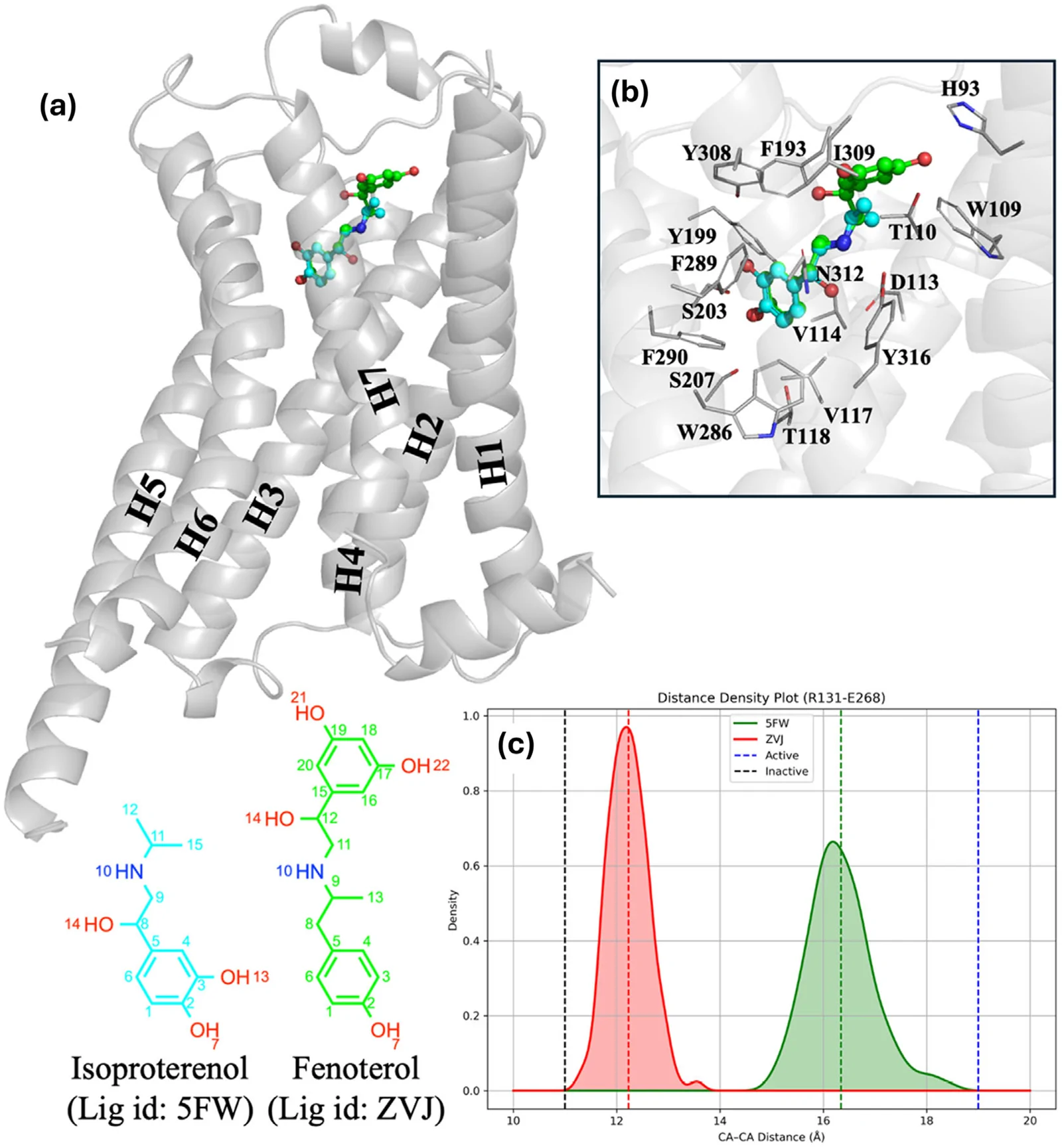
Differences in β_2_AR structure. (a) Crystal structure with ISO (CPK with cyan carbons) with FENO (CPK model with green carbons) overlaid on ISO in the orthosteric pocket. Ligand chemical structures with atom numbers are shown. (b) Close-up view of the orthosteric site with the ligands and the side chains of the residues defining the orthosteric binding site (<3.5 Å from the ligands). (c) Ionic-lock alpha carbon (Arg131^3.50^-Glu268^6.30^) distance measured from the MD simulations. The black and blue dashed line indicates inactive (~11 Å) and active (~19 Å) states collected from the crystal conformations. The green and red dashed lines represent the average distances and the density distributions of the ionic-lock distances from MD simulations in the presence of ISO (5FW) and FENO (ZVJ). FENO: fenoterol; ISO: isoproterenol.

To investigate the mechanism for the lack of effect of AMs on fenoterol-induced Gαs-cAMP-PKA signalling, we performed molecular dynamics (MD) simulations of β_2_ARs bound to two agonists, isoproterenol and fenoterol (Figure 8). We focused on conformational changes that influence the accessibility of the novel allosteric binding site. To compare global conformational stability, we analysed root mean square deviation (RMSD) profiles of β_2_AR conformations in the presence of isoproterenol or fenoterol. RMSD analyses revealed distinct conformational trajectories relative to the initial inactive-state structure (Figure S7), with the isoproterenol-bound receptor remaining closer to the active-state conformation and exhibiting greater stability over time compared to the fenoterol-bound receptor (Figure S7). Structural overlays of the final simulation pose relative to the active β_2_AR crystal structure (Figure S8) further demonstrated orthosteric ligand-receptor binding. The RMSD plot and structural comparison show that the isoproterenol (5FW) bound β_2_AR is less deviated from its initial crystal conformation and remains stable during simulation (Figure S8C) compared to the fenoterol (ZVJ) bound β_2_AR (Figure S8D), as reflected in the RMSD plot (Figure S7). We observe that the TM6 and TM5 helices have moved significantly in the presence of fenoterol.

The ionic lock is a key salt bridge between conserved residues in GPCRs. Disruption of the conserved ionic lock is a hallmark of β_2_AR activation and was quantified by measuring the Cα distance between Cα atoms of Arg131^3.50^ and Glu268^6.30^. MD simulations in the inactive β_2_AR crystal structure (PDB: 5X7D) revealed an increase in distance of ~11 to 16.4 Å upon isoproterenol binding, consistent with a fully active receptor conformation. In contrast, fenoterol produced a more limited displacement of the ionic lock distance (12.3 Å), positioning the receptor closer to the inactive state than to the fully activated conformation stabilized by isoproterenol (Figure 8c, Table S6A). To directly assess the changes in geometry of the allosteric pocket shape, we quantified the distances between Cα atoms of residues Arg131^3.50^, Phe223^5.62^, Tyr274^6.36^ and Arg328^7.55^ on TM3, TM5, TM6 and TM7, respectively (Table S6B). In isoproterenol-bound simulations, TM6 remained in an outward, active-like position, preserving an open and well-defined allosteric pocket. By contrast, fenoterol binding promoted inward repositioning of TM6 and associated helices, resulting in a more constricted pocket geometry and reduced accessibility relative to the isoproterenol-stabilized state (Figures 8 and S9).

Collectively, these in silico analyses indicate that fenoterol stabilizes β_2_AR conformations distinct from those induced by isoproterenol, leading to an altered allosteric pocket shape that is structurally less permissive for the AMs binding, consistent with the absence of modulation observed experimentally.

### 3.10 | Allosteric modulators selectively modulate Gαs-cAMP-PKA signalling and bronchodilation without affecting the β-arrestin pathway

**FIGURE 9:**
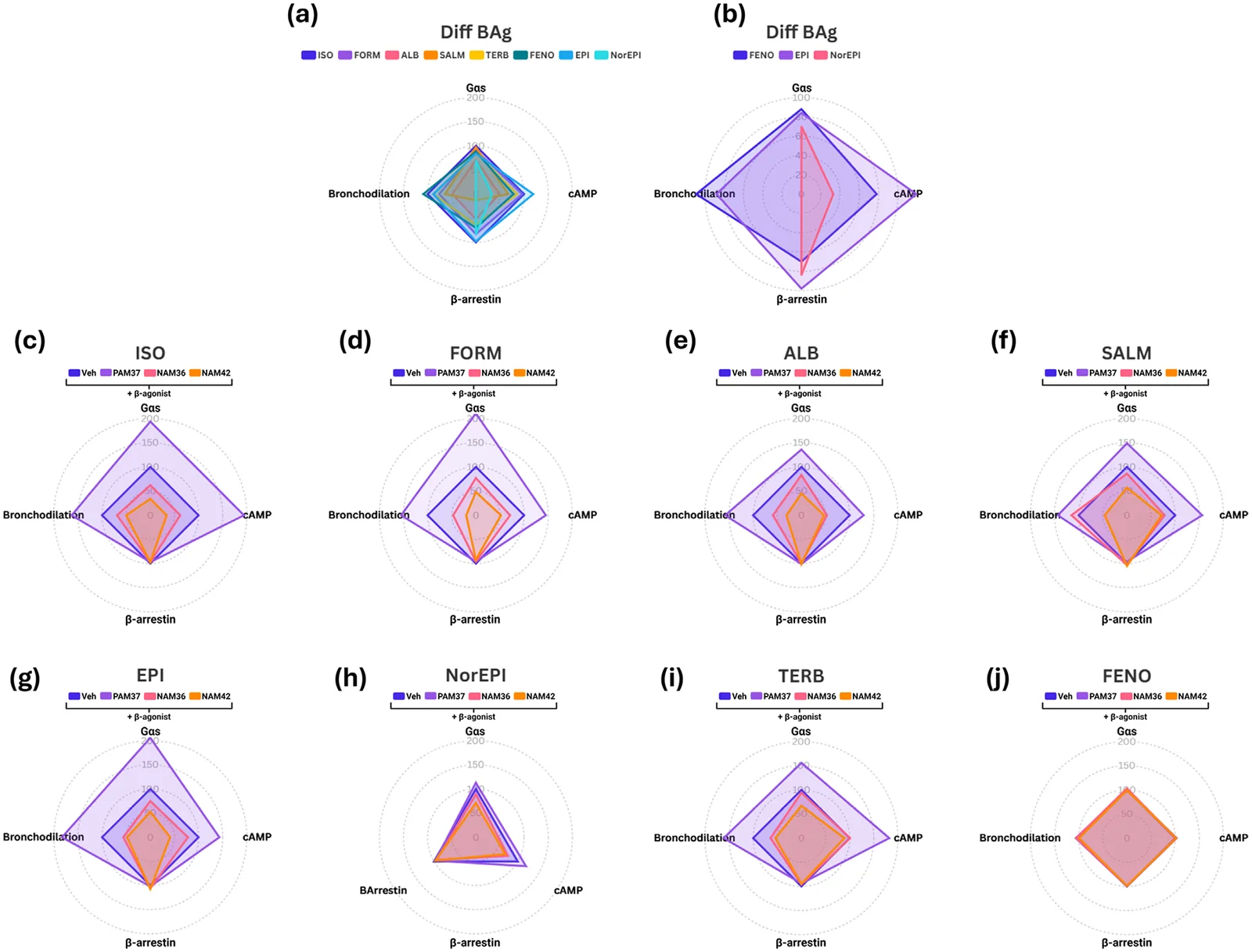
Comparative bias profiling of β_2_AR AMs reveals β-agonist-specific modulation of Gαs-cAMP-PKA signalling and bronchodilation. To integrate signalling and functional data, radar plots were generated for each β-agonist summarizing the effects of AMs on Gαs recruitment, cAMP generation, β-arrestin recruitment and bronchodilation. Data for E_max_ values were compiled from dose–response curves (Figures 1, 3, 4, 6 and S1) and normalized to each β-agonist’s vehicle control response. Radar plots of (a and b) unmodulated β-agonist responses (β-agonist only) and (c–j) in the presence of 100-nM AMs illustrate the differential effects of PAM37, NAM36 and NAM42 on each signalling parameter. PAM37 enhanced and NAM36 and NAM42 attenuated Gαs-cAMP-PKA signalling and bronchodilation for (c–j) ISO, FORM, ALB, SALM, EPI, NorEPI and TERB, but not when treated with FENO (j). ALB: albuterol; EPI: adrenaline; FENO: fenoterol; FORM: formoterol; ISO: isoproterenol; NAM: negative allosteric modulator; NorEPI: noradrenaline; PAM: positive allosteric modulator; SALM: salmeterol; TERB: terbutaline.

Next, we generated comparative radar plots using E_max_ values obtained from β_2_AR-Gαs coupling, cAMP production, β-arrestin recruitment and bronchodilatory response studies (Figure 9). These plots reveal that PAM37 consistently enhances Gαs-cAMP signalling and bronchodilation across multiple β-agonists (isoproterenol, formoterol, albuterol, salmeterol, adrenaline, terbutaline and noradrenaline) (Figure 9c–j). Conversely, NAM36 and NAM42 broadly inhibit Gαs-cAMP signalling and bronchodilation (Figure 9c–j). None of the AMs affected β-agonist-mediated β-arrestin recruitment, suggesting that the AMs exhibit signalling and functional bias. Notably, the degree to which AMs modulated Gαs signalling differed among the agonists, including no effect on fenoterol, suggesting a ligand-dependent allosteric effect of AMs.

## 4 | DISCUSSION

The current study establishes that the AMs exhibit clear agonist-specific activity, potentiating or inhibiting the Gαs-cAMP-PKA pathway for multiple agonists, with fenoterol being uniquely resistant to allosteric modulation. Importantly, this modulation remained selectively biased towards the Gαs pathway with no effect on agonist-induced β-arrestin recruitment or β-arrestin-mediated signalling, aligning with our previous results performed during compound screening (Shah et al., 2022). Furthermore, PAM37 potentiated the bronchodilatory response of multiple β-agonists ex vivo and in vivo, underscoring the therapeutic potential of Gαs-biased AMs.

Recent advances in GPCR structural biology and molecular modelling have aided the development of new β_2_AR ligands with diverse pharmacological properties (Ahn et al., 2018, 2023; De Pascali et al., 2022; Ippolito et al., 2023; Shah et al., 2022; Shah & Deshpande, 2025), including ligands that have the ability to selectively activate/inhibit Gαs or β-arrestin signalling and functions. Such ‘biased’ β_2_AR ligands include orthosteric agonists (which bind to conventional orthosteric binding sites) and AMs (which bind to a different site than orthosteric ligands) (Shah & Deshpande, 2025). Biased β_2_AR ligands, including agonists and allosteric modulators, that either positively skew receptor signalling towards the Gαs-cAMP-PKA pathway or inhibit the β-arrestin pathway are conceptually attractive for the management of asthma (Ahn et al., 2018, 2023; De Pascali et al., 2022; Kim et al., 2021; Rankovic et al., 2016; Shah & Deshpande, 2025). This hypothesis is supported by multiple signalling and functional studies in ASM cells and airways showing that β_2_AR-mediated effects of β-agonists are Gαs-PKA-dependent (Misior et al., 2008; Morgan et al., 2014; Yan et al., 2011). Conversely, β-arrestin-mediated desensitization causes a ~20% decrease in β_2_AR signalling, as well as enhances asthma pathology in murine models (Deshpande et al., 2008; Nguyen et al., 2009; Penn et al., 1998; Walker et al., 2003).

Multiple biased orthosteric β-agonists, including C1-S, ractopamine, dobutamine and higenamine, and AMs, including Cmpd-6, Cmpd-43 and DFPQ, have been described in the literature (Ahn et al., 2018; De Pascali et al., 2022; Ippolito et al., 2023; Kim et al., 2021; Shah et al., 2022; Shah & Deshpande, 2025). Similar to PAM37, NAM36 and NAM42 described here, Cmpd-6 and DFPQ are AMs that lack intrinsic activity and thus require an orthosteric ligand to modulate signalling. While these AMs promote ASM relaxation or reduce receptor desensitization, their signalling profiles differ: Some exhibit balanced modulation of Gαs and β-arrestin pathways, and others exhibit selective β-arrestin inhibition, offering distinct therapeutic advantages depending on the context. Specifically, DFPQ functions as a β-arrestin-biased NAM, attenuating β-arrestin recruitment while preserving Gαs signalling, thereby preventing receptor desensitization and sustaining bronchodilator responsiveness. Furthermore, translating the signalling profiles into functional outcomes in physiologically relevant models remains a critical step. The AM Cmpd-6 has demonstrated PAM activity for signalling induced by isoproterenol, albuterol, salmeterol and fenoterol in cell-based assays. The bronchodilatory effect of Cmpd-6 was tested only against a single agonist, fenoterol (Ahn et al., 2023). In the current study, we characterize the activity of PAM37 across eight distinct β-agonists using both ex vivo (mPCLS) and in vivo lung function measurements to establish its translational potential. Furthermore, PAM37 and NAM36 and NAM42 bind to a novel allosteric site defined by residues Arg131^3.50^, Tyr219^5.58^ and Phe282^6.44^ in a pocket located deep within the transmembrane, and other known AMs bind to sites closer to the intracellular face or membrane interface (Ahn et al., 2018; Shah et al., 2022; Shah & Deshpande, 2025). Most importantly, our AMs exhibit a unique bias profile. Unlike balanced modulators (such as Cmpd-6) or β-arrestin-specific modulators (such as DFPQ), PAM37, NAM36 and NAM42 selectively alter the Gαs-cAMP-PKA signalling pathway without affecting the β-arrestin pathway. Importantly, a PAM that selectively enhances the Gαs-cAMP-PKA pathway without affecting β-arrestin recruitment is a desirable therapeutic alternative. This approach could lower β-agonist doses to achieve similar or superior clinical outcomes, thereby reducing the chances of β-arrestin-mediated receptor desensitization and signalling.

By examining endogenous catecholamines and various classes of clinically used β-agonists, we demonstrated that the AMs could modulate β_2_AR signalling across diverse pharmacological profiles. A major strength of this study is the comprehensive comparison of these eight distinct clinically relevant and endogenous β-agonists across multiple experimental outcomes. The chosen β-agonists differed in their intrinsic efficacy (full or partial agonists) and duration of action (short-acting β-agonists [SABAs] or long-acting β-agonists [LABAs]). Our results indicate that the AMs modulate signalling and functional outcomes irrespective of the efficacy and duration of action of the β-agonists. While this broad activity demonstrates the versatility and therapeutic potential of these AMs, the lack of signalling modulation with fenoterol and the differential effects with terbutaline reveal an additional layer of β-agonist-dependent regulation of AM efficacy. MD simulations further revealed β-agonist-dependent changes in allosteric site geometry and accessibility.

β-Agonists induce a spectrum of highly dynamic ligand-dependent active-state-like β_2_AR conformations, characterized by the outward movement of TM6, closure of extracellular loop 2 (ECL2), a twist in TM7 and intracellular loop 2 (ICL2) stabilization (Bhattacharya et al., 2008; Calderon et al., 2023; Dror et al., 2011a; Liang et al., 2017; Liu et al., 2012; West et al., 2011). The extent and combination of these changes account for differences in efficacy, potency and signalling bias (Bhattacharya et al., 2008; Liu et al., 2012; Plazinski et al., 2025; Tikhonova et al., 2013). For instance, full or near full agonists such as isoproterenol and adrenaline stabilize a fully active conformation via strong polar interactions between Asp113^3.32^ (TM3), Ser203^5.42^, Ser204^5.43^ and Ser207^5.46^ (TM5) and Asn293^6.55^ (TM6), inducing an extensive outward shift of TM6 and a closed ECL2 loop, enabling G protein engagement. In contrast, partial agonists, including albuterol and salmeterol, engage fewer polar interactions due to rotameric shift of Ser204^5.42^ and reduced H-bonding to Asn293^6.55^, resulting in submaximal receptor activation (Bhattacharya et al., 2008; Lakkaraju et al., 2016; Liu et al., 2012; Plazinski et al., 2025; Shah & Deshpande, 2025; Tikhonova et al., 2013; Yang et al., 2021). Additional structural determinants of G protein bias have been linked to the outward shift of TM7 and the open conformation of intracellular loop 3 (ICL3) in addition to changes in TM6 and ECL2 (Chen et al., 2022; Plazinski et al., 2025; Tikhonova et al., 2013). The structural differences in β-agonist-dependent activation of β_2_ARs are consistent with our Gαs-cAMP-PKA signalling data (Figures 1a, 4a, 6a, and S1A). We observed that fenoterol-induced Gαs signalling and bronchodilation are uniquely resistant to modulation by PAM37, NAM36 and NAM42. Comparing the structures of fenoterol and isoproterenol reveals similarities in the core structure of β-agonists. However, fenoterol is structurally more elaborate, with an added methyl group and a phenyl ring (compared to isoproterenol), which could account for unique conformational changes in the β_2_AR induced by fenoterol. The roles of other structural features of fenoterol and its stereoisomers need to be comprehensively established in future studies. Importantly, our radioligand binding assays confirmed that the inability of PAM37, NAM36 and NAM42 to modulate fenoterol-induced signalling is not due to AM-induced reduction in fenoterol binding affinity. Instead, the mechanism of this resistance lies in the distinct conformational state stabilized by fenoterol. Using in silico modelling, we demonstrate changes in the β_2_AR structure upon binding with isoproterenol and fenoterol. Specifically, we identify that fenoterol induces a pronounced inward displacement of TM6 relative to a fully active β_2_AR conformation, compared with isoproterenol. This reduces the accessibility of the allosteric site to AMs when fenoterol is bound to the orthosteric site. Notably, similar differences with fenoterol have been observed: fenoterol induces a unique conformation characterized by a large decrease in the distance between ICL2 and the C-terminus, in contrast to the increases observed with formoterol and the minor shifts induced by albuterol and salmeterol (Swift et al., 2007). This distinctive conformational state hinders the availability of the allosteric pocket, preventing the binding of PAM37, NAM36 and NAM42. Collectively, this renders fenoterol and β-agonists that induce a fenoterol-like conformational change as poor candidates for this specific class of AMs.

An interesting finding is that the rank order of modulation by the AMs differed across signalling and functional assays. For example, PAM37 produced the greatest enhancement of Gαs-coupling with formoterol but showed a greater effect on bronchodilation with adrenaline. These apparent discrepancies likely reflect the complexity of GPCR signal transduction. A possible explanation for the differences in the rank order of AMs across assays is compartmentalization of signalling, as localized pools of cAMP and downstream effectors in HEK293 or HASM cells or tissues could yield divergent responses. Additionally, the relative expression levels of proteins (β_2_AR and its downstream effectors) play a key role in receptor activation and nonlinear signal amplification. Another contributing factor is that the concentration ranges used in each assay differ, which may differentially capture the maximal efficacy of each agonist across assays. Together, these considerations likely account for assay-dependent differences we observed rather than a uniform magnitude rank order.

Beyond signalling and structural changes, our detailed kinetic analyses provided nuanced kinetic profiles of β-agonist-dependent Gαs-coupling dynamics that are reshaped by AMs. PAM37 enhanced, whereas NAM36 and NAM42 attenuated, the rate and magnitude of receptor activation. These effects highlight that modulation by AMs not only alters signalling amplitude but also alters the temporal kinetics of receptor activation, providing an additional mechanism to explain the differential modulation observed across β-agonists. To contextualize our findings, we generated comparative radar plots summarizing the modulation of Gαs coupling, cAMP generation, β-arrestin recruitment and bronchodilation across all β-agonists tested. The analysis reveals selective modulation of the Gαs-cAMP-PKA pathway by our AMs. Importantly, AMs do not affect β-arrestin-ERK signalling, indicating that β-agonist-mediated receptor internalization and desensitization are not altered. While receptor internalization and desensitization were not directly assessed in ex vivo/in vivo models, the augmentation of bronchodilation has significant implications. Notably, β_2_AR desensitization is often observed at higher concentrations of β-agonist. In the presence of PAM37, β-agonists were more efficacious than β-agonists alone, thereby requiring lower doses to achieve optimal bronchodilation, which potentially decreases the likelihood of receptor desensitization and internalization.

In summary, these findings collectively demonstrate the therapeutic potential of PAM37 to enhance the effectiveness of existing therapeutics for obstructive airway diseases. PAM37 promotes, and NAM36 and NAM42 attenuate, Gαs-cAMP-PKA signalling and β-agonist-induced bronchodilation in an agonist-dependent manner, without affecting β-arrestin-ERK signalling, underscoring their structural, functional, and translational relevance. Future studies will assess the effects of chronic AMs use, examine their pharmacokinetic properties, and their impact on β_2_AR internalization and desensitization in vivo.

## Supplementary Material

supplemental information

Additional supporting information can be found online in the Supporting Information section at the end of this article.

## Figures and Tables

**TABLE 1:** E_max_ and pEC_50_ for Gαs coupling to the β_2_AR, and effects of negative and positive allosteric modulators (NAMs and PAMs)

A				
	Gαs-recruitment—E_max_			
	Vehicle	PAM37	NAM36	NAM42
ISO	101.6 ± 2.1	187.4 ± 6.9*	65.9 ± 2.5*	36.8 ± 1.4*
FORM	89.9 ± 2.8	179.3 ± 7.5*	69.3 ± 2.7*	45.9 ± 1.1*
ALB	69.9 ± 4.4	93.8 ± 5.1*	49.1 ± 2.7*	28.4 ± 1.3*
SALM	94.3 ± 5.3	143.5 ± 7.9*	71.1 ± 3.2*	50.8 ± 3.1*
TERB	82.5 ± 5.7	129.2 ± 5.2*	77.3 ± 6.0	55.3 ± 2.9*
EPI	85.6 ± 5.0	168.3 ± 7.6*	63.8 ± 3.0*	45.8 ± 1.7*
NorEPI	67.3 ± 4.5	81.0 ± 4.2*	54.3 ± 2.5*	42.4 ± 2.3*
FENO	84.2 ± 6.6	83.0 ± 4.4	86.3 ± 4.4	83.4 ± 3.0
B				
	Gαs-recruitment—pEC_50_			
	Vehicle	PAM37	NAM36	NAM42
ISO	6.4 ± 0.1	6.0 ± 0.0	6.5 ± 0.2	6.3 ± 0.2
FORM	6.1 ± 0.0	6.0 ± 0.0*	6.2 ± 0.0*	6.7 ± 0.0*
ALB	7.0 ± 0.0	6.8 ± 0.0*	5.0 ± 0.0*	5.3 ± 0.0*
SALM	7.3 ± 0.0	6.7 ± 0.0*	5.1 ± 0.0*	5.4 ± 0.0*
TERB	7.4 ± 0.0	7.4 ± 0.0*	7.4 ± 0.0	7.5 ± 0.0*
EPI	6.9 ± 0.0	6.7 ± 0.0*	6.6 ± 0.0*	7.0 ± 0.0*
NorEPI	6.3 ± 0.0	6.4 ± 0.0*	5.2 ± 0.0*	5.1 ± 0.0*
FENO	7.4 ± 0.0	7.4 ± 0.0	7.4 ± 0.0	7.4 ± 0.0

*Note*: (A) E_max_ and (B) pEC_50_ calculated from dose–response curves from cells stimulated with increasing concentrations of β-agonists in the presence of vehicle or 100-nM AMs.

* P < 0.05 β-agonist versus AMs + β-agonist.

## Data Availability

All data needed to evaluate the conclusions are present in the manuscript or the Supporting Information. The data that support the findings of this study are available from the corresponding author upon reasonable request. Some data may not be made available because of privacy or ethical restrictions.

## What is already known

- β_2_-adrenoceptor agonists differ in their signaling and bronchodilation profile. Diverse β-agonists stabilize unique receptor conformations.
- Selective augmentation of beneficial β_2_AR-Gαs without increases in β-arrestin signaling enhances the β-agonists’ therapeutic efficacy.

## What this study adds

- PAM37 amplifies beneficial Gαs-signaling across commonly used β-agonists without affecting β-arrestin signaling.
- The pharmacological features of β_2_-adrenoceptor allostery are governed by the specific orthosteric ligand.

## Clinical Significance

- PAM37 selectively enhances bronchodilation by clinically relevant β-agonists, enabling precision pharmacotherapy for obstructive airway diseases.
- Agonist-dependent allosteric modulation forms the mechanistic basis for future drug discovery efforts.

## Abbreviations:

[^3^H]DHA: radiolabelled dihydroalprenolol
ALB: albuterol
AM: allosteric modulator
ASM: airway smooth muscle
β-agonist: beta-adrenoceptor agonist
BRET: bioluminescence resonance energy transfer
EPI: adrenaline
ERK: extracellular signal-regulated kinase 1/2
FEN: fenoterol
FORM: formoterol
GRK: G protein-coupled receptor kinase
Gα: alpha subunit of G protein
Gs/Gαs: stimulatory G protein alpha subunit
HEK293: human embryonic kidney 293 cells
ISO: isoproterenol
MD: molecular dynamics
mPCLS: murine precision-cut lung slices
NAM: negative allosteric modulator
NAM36: negative allosteric modulator compound 36
NAM42: negative allosteric modulator compound 42
NorEPI: noradrenaline
PAM: positive allosteric modulator
PAM37: positive allosteric modulator compound 37
PCA: principal component analysis
PCLS: precision-cut lung slices
RMSD: root mean square deviation
SALM: salmeterol
TERB: terbutaline
TM: transmembrane
VASP: vasodilator-stimulated phosphoprotein
