# Supplementary material for: Agonist-specific conformational dynamics at the β2-adrenoceptor dictate allosteric modulation of Gαs signalling and bronchodilation
Source: Br J Pharmacol. Author manuscript; Available in PMC 2026 Sep 17. (PMC13583984; doi:10.1111/bph.70513)
Supplement: Supplementary file 1 [file NIHMS2209339-supplement-supplemental_information.pdf]

**SUPPLEMENTARY INFORMATION**

# Agonist-specific conformational dynamics at the β$_2$-adrenoceptor dictate allosteric modulation of Gαs signalling and bronchodilation

Sushrut D. Shah[1], Joshua Richard[1], Suvankar Ghosh[2], Jillian Jerwick[1], Joice T. Gavali[1], Raymond B. Penn[1], Alexander D. MacKerell, Jr.[2], and Deepak A. Deshpande[1#]

[1]Center for Translational Medicine, Jane and Leonard Korman Respiratory Institute, Thomas Jefferson University, Philadelphia, PA 19107

[2]Department of Pharmaceutical Sciences, School of Pharmacy, University of Maryland, Computer-Aided Drug Design Center, University of Maryland, Baltimore, MD 21201

**Figure S1**

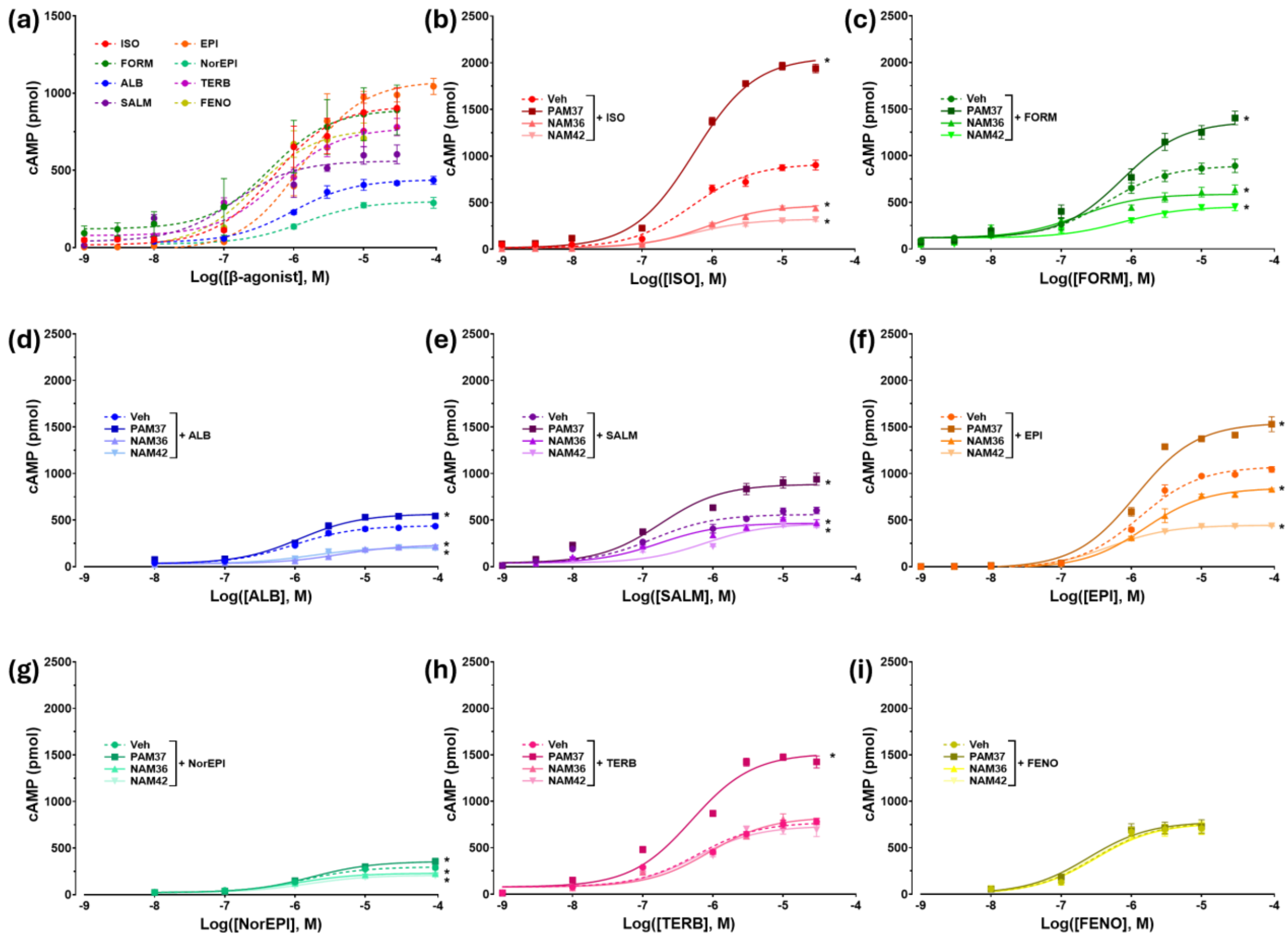


**Figure S1. Modulation of different β-agonist-induced cAMP generation in HEK293 cells expressing human β$_2$AR by the AMs.** HEK293 cells transfected with human β$_2$AR were plated in 24-well plates and stimulated with 100 nM AMs or vehicle in the presence of increasing concentrations of various β-agonists for 10 minutes. Generation of cAMP was measured by ELISA, and dose-response curves were generated using a non-linear logistic curve from multiple experiments. Dose-response curves of (A) various β-agonists alone or (B-I) in the presence of 100 nM AMs. PAM37 augmented and NAM36 and NAM42 inhibited cAMP generation induced by (B) ISO, (C) FORM, (D) ALB, (E) SALM, (F) EPI, and (G) NorEPI, but had variable effects with (H) TERB (PAM augmented, but NAMs did not inhibit) and no effect with (I) FENO. N = 4-8. Statistical analysis was performed using a two-way ANOVA with a Bonferroni post hoc test. *$P \leq 0.05$ β-agonist vs AMs+β-agonist. PAM: Positive Allosteric Modulator; NAM: Negative Allosteric Modulator; ISO: isoproterenol; FORM: formoterol; ALB: albuterol; SALM: salmeterol; EPI: adrenaline; NorEPI: noradrenaline; TERB: terbutaline; FENO: fenoterol.

## Figure S2

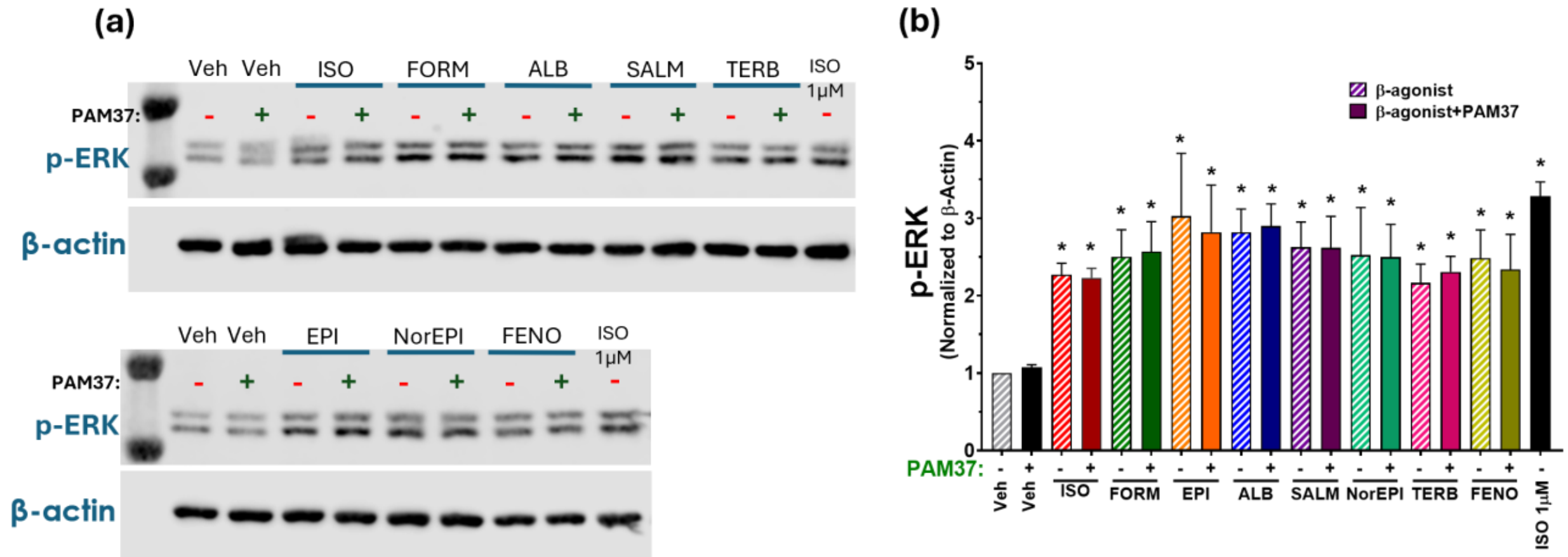


**Figure S2. PAM37 does not affect β-agonist-induced ERK1/2 phosphorylation in primary human ASM cells.** Cells were plated in 24-well plates and stimulated with 100 nM PAM37/vehicle in the presence of various β-agonists for 5 minutes. β-agonists were used at the following concentrations: 100 nM isoproterenol (ISO), 100 nM formoterol (FORM), 100 nM albuterol (ALB), 100 nM salmeterol (SALM), 100 nM terbutaline (TERB), 100 nM adrenaline (EPI), 1 µM noradrenaline (NorEPI), and 1 µM fenoterol (FENO). GRK-β-arrestin-ERK1/2 signalling was examined by measuring ERK1/2 phosphorylation by western blotting. (A) Representative western blot of various β-agonists in the presence of vehicle or 100 nM PAM37. (B) Band intensities of phosphor-ERK1/2 were quantified and normalized to β-actin. PAM37 did not affect β-agonist-induced ERK1/2 phosphorylation. n = 4-5. PAM: Positive Allosteric Modulator; ISO: isoproterenol; FORM: formoterol; ALB: albuterol; SALM: salmeterol; EPI: adrenaline; NorEPI: noradrenaline; TERB: terbutaline; FENO: fenoterol.

**Figure S3**

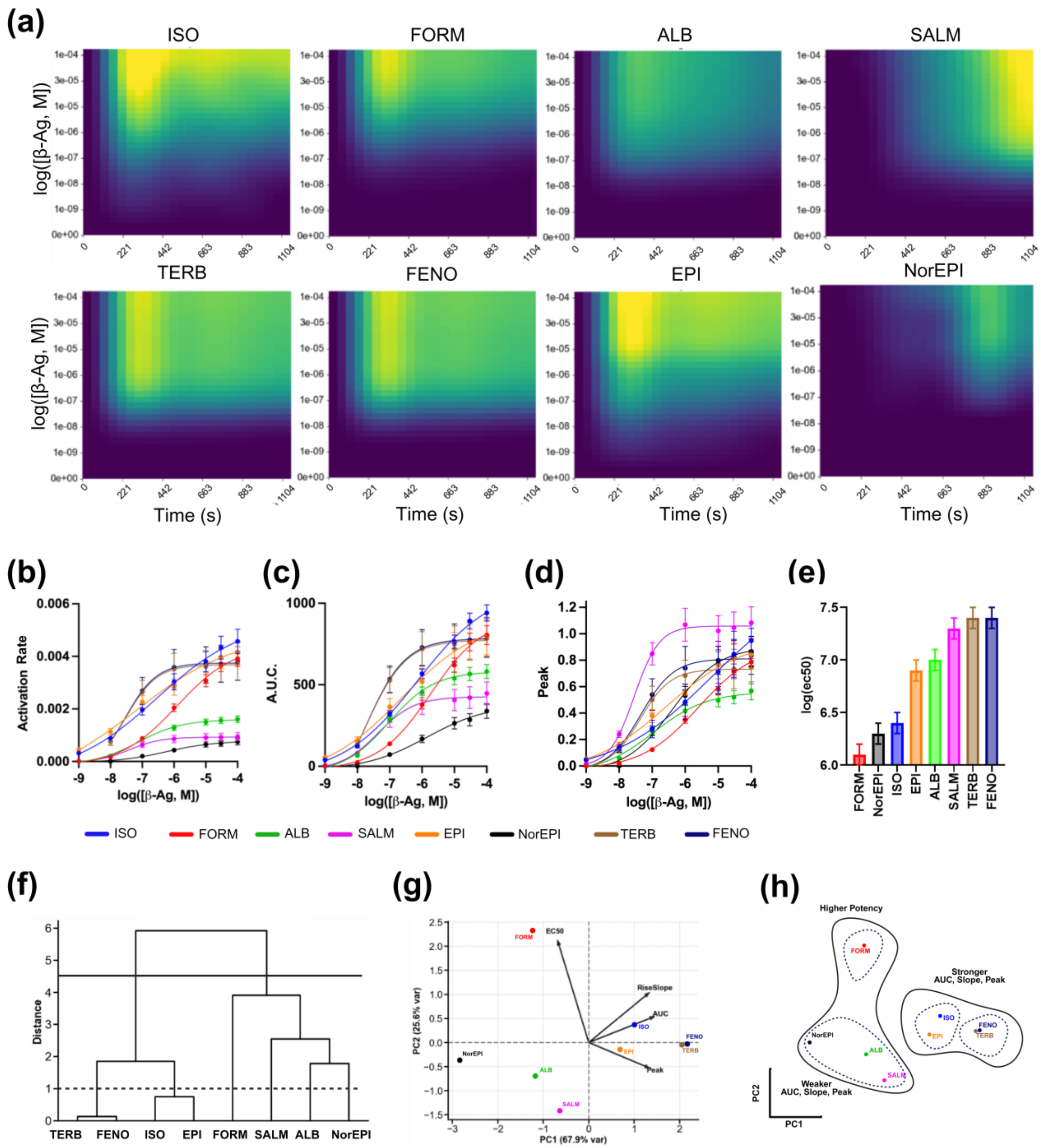


**Figure S3. Dose-response kinetics of β-agonist–induced Gαs coupling to the β$_2$AR.** In-depth analyses of β-agonist-induced Gαs-coupling were performed to assess differences in the kinetic profile of Gαs activation by different β-agonists. (A) Contour plots depict Gαs recruitment traces across doses of eight β-agonists, expressed as percent of the maximal ISO response. (B-E) Quantitative analyses of the kinetic and dose–response parameters derived from the traces, including (B) activation rate, (C) area under the curve, (D) maximal peak signal, and (E) log(EC$_{50}$) values. (F) Hierarchical clustering dendrogram depicts

the relationships among agonists based on AUC, $EC_{50}$, Emax, and Hill slope. (G) Principal component analysis (PCA) was used to classify the β-agonists based on their characteristics. A biplot with the contribution of each feature to map β-agonist variance. Arrow direction denotes correlation with agonist placement, and arrow length indicates relative contribution. (H) Diagram depicting computed strict and relaxed clusters of agonists in PCA space based on features. PAM: Positive Allosteric Modulator; NAM: Negative Allosteric Modulator; AUC: Area under the curve; ISO: isoproterenol; FORM: formoterol; ALB: albuterol; SALM: salmeterol; EPI: adrenaline; NorEPI: noradrenaline; TERB: terbutaline; FENO: fenoterol.

## Figure S4

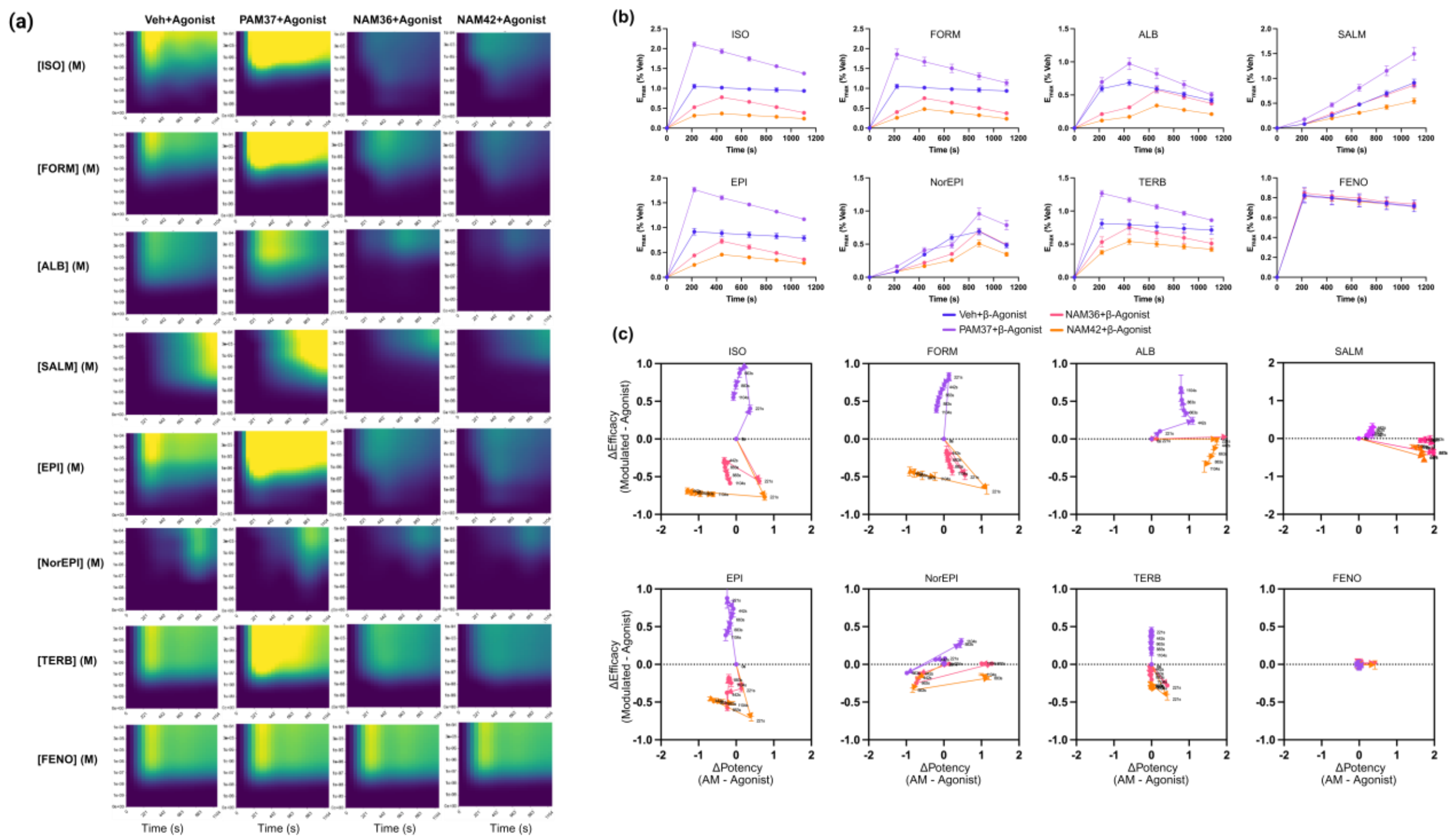


**Figure S4. Temporal analysis reveals dynamic modulation of β-agonist efficacy and potency by AMs. (**A) Contour plots of β-agonist–induced Gαs coupling to $\beta_2$AR over time and dose (first column), and the modulation of this signal by PAM37 (second column), NAM36 (third column), and NAM42 (fourth column). (B) Temporal evolution of Emax for each β-agonist in the presence of vehicle or AMs. (C) Vector plots highlight the effects of AMs on β-agonist potency and efficacy over time. Symbols denote individual time points with the arrows indicating the progression of time. PAM: Positive Allosteric Modulator; NAM: Negative Allosteric Modulator; AUC: Area under the curve; ISO: isoproterenol; FORM: formoterol; ALB: albuterol; SALM: salmeterol; EPI: adrenaline; NorEPI: noradrenaline; TERB: terbutaline; FENO: fenoterol.

**Figure S5**

**(a)** Peak

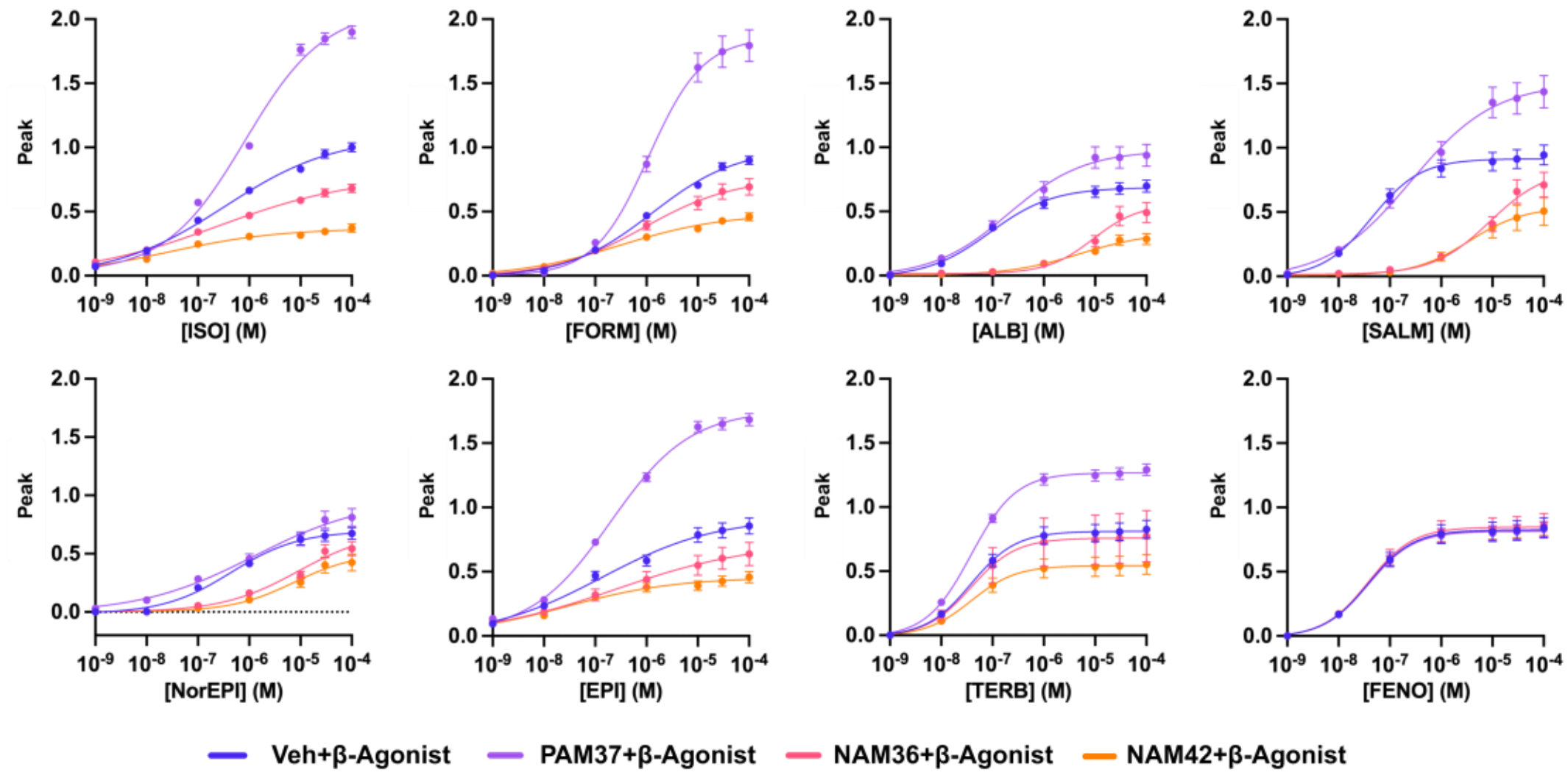


**(b)** Activation Slope (k)

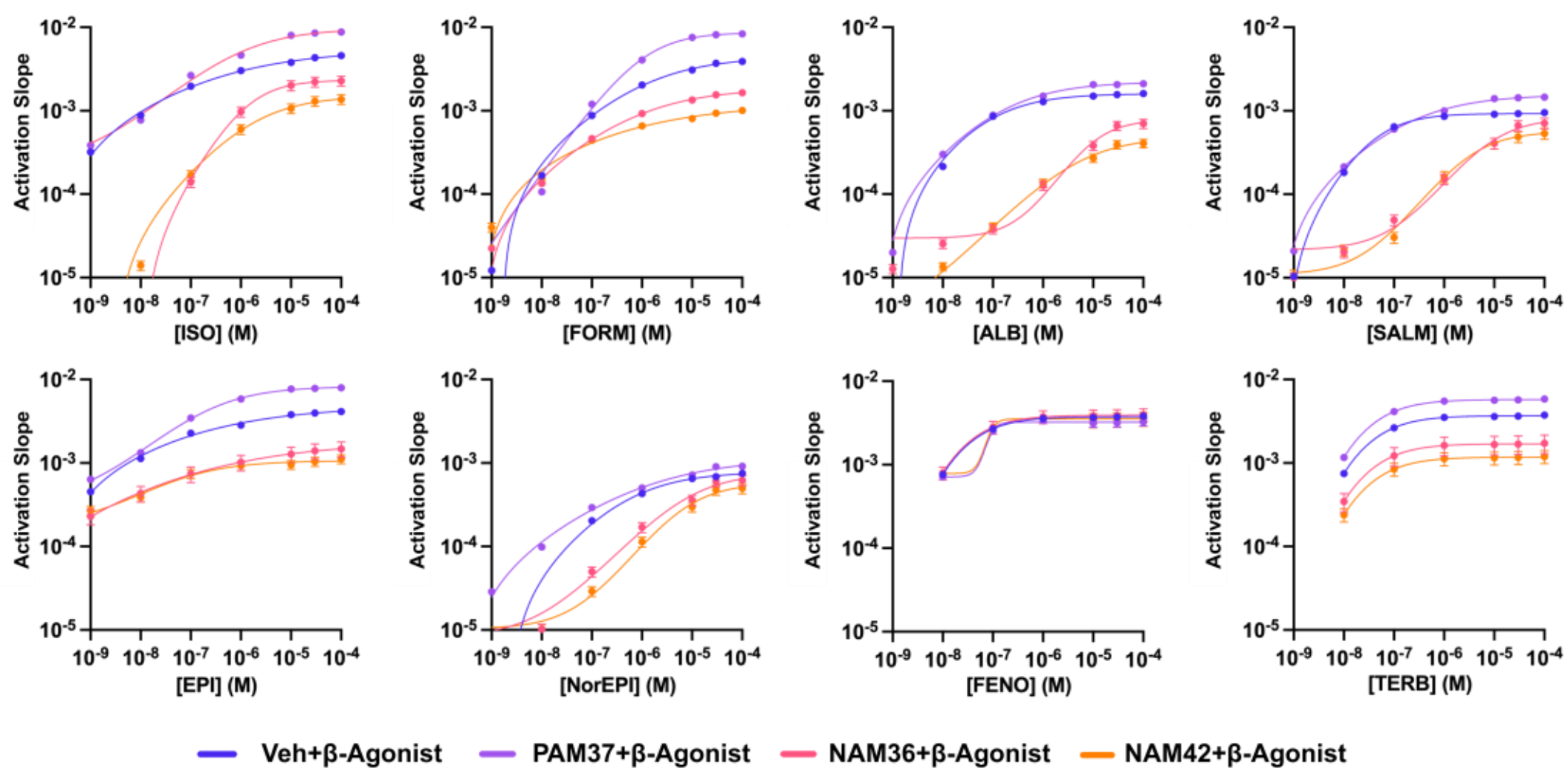


**(c)** A.U.C.

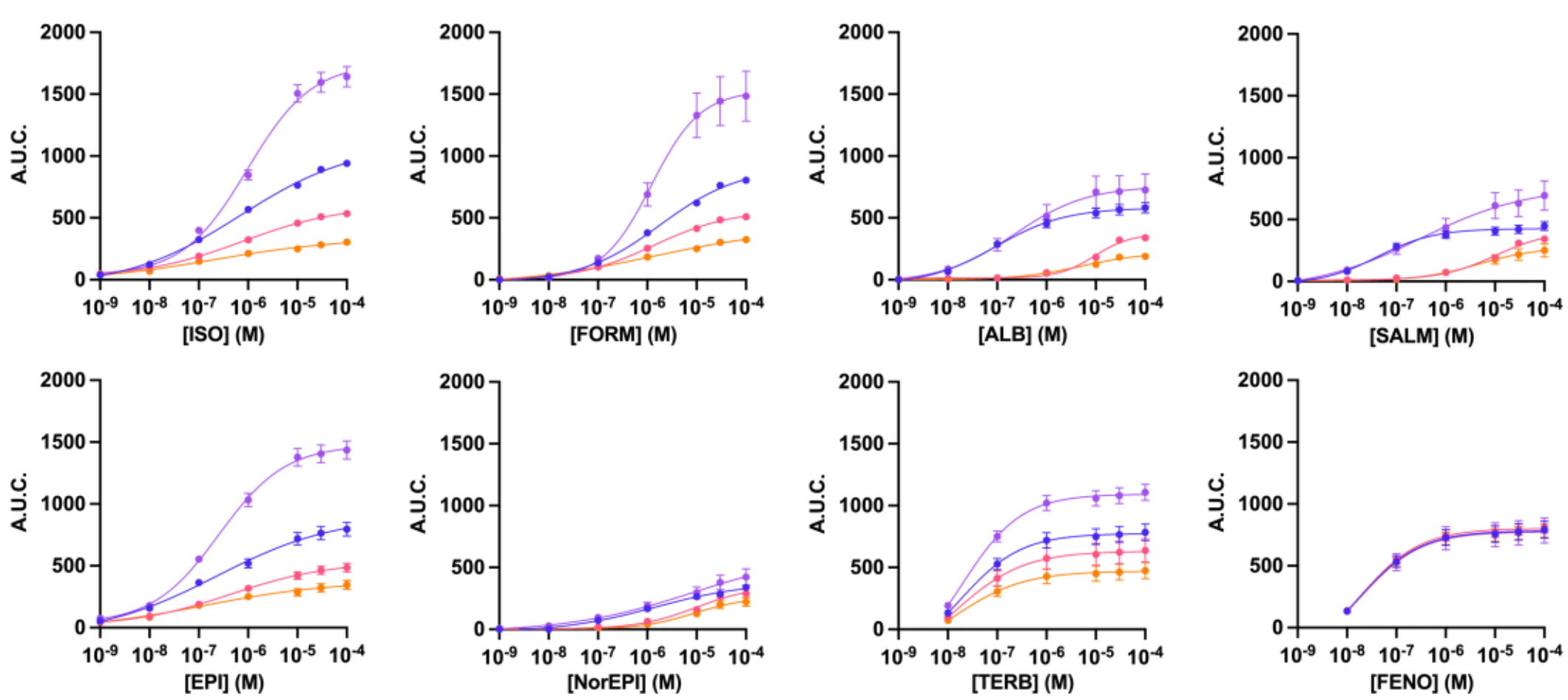

**Figure S5: AMs differentially modulate the magnitude, rate, and total extent of β-agonist-induced Gαs-coupling with $\beta_2$AR.** (A-C) Quantification of AM's effects on kinetic features of β-agonist–induced Gαs coupling to $\beta_2$AR signal across doses, including (A) maximal peak signal, (B) activation rate, and (C) area under the curve (AUC). PAM: Positive Allosteric Modulator; NAM: Negative Allosteric Modulator; AUC: Area under the curve; ISO: isoproterenol; FORM: formoterol; ALB: albuterol; SALM: salmeterol; EPI: adrenaline; NorEPI: noradrenaline; TERB: terbutaline; FENO: fenoterol.

**Figure S6**

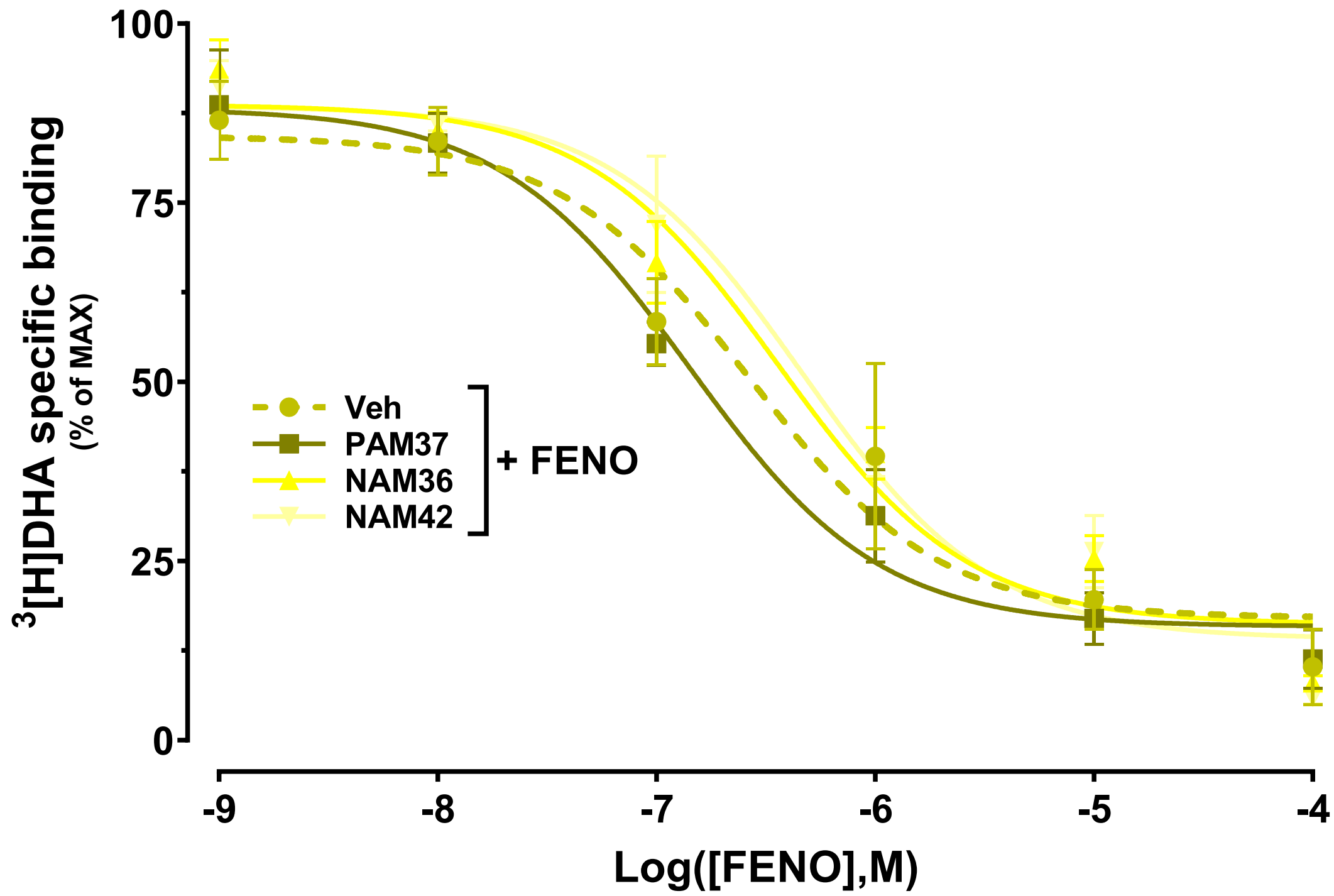


**Figure S6: AMs do not alter fenoterol binding affinity to the β$_2$AR.** Competitive radioligand binding assays were performed using membranes from HEK293 cells expressing human β$_2$AR. Membranes were incubated with 3 nM [$^3$H]-DHA and increasing concentrations of fenoterol in the presence of vehicle or 100 nM AMs. Data is expressed as the percentage of the maximum [$^3$H]-DHA-specific binding. Dose-response curves were generated using non-linear regression from multiple experiments. PAM37, NAM36, and NAM42 did not alter the binding affinity of fenoterol to the β$_2$AR. n=3-4. [$^3$H]-DHA: [$^3$H]-dihydroalprenolol; PAM: Positive Allosteric Modulator; NAM: Negative Allosteric Modulator; FENO: fenoterol.

**Figure S7**

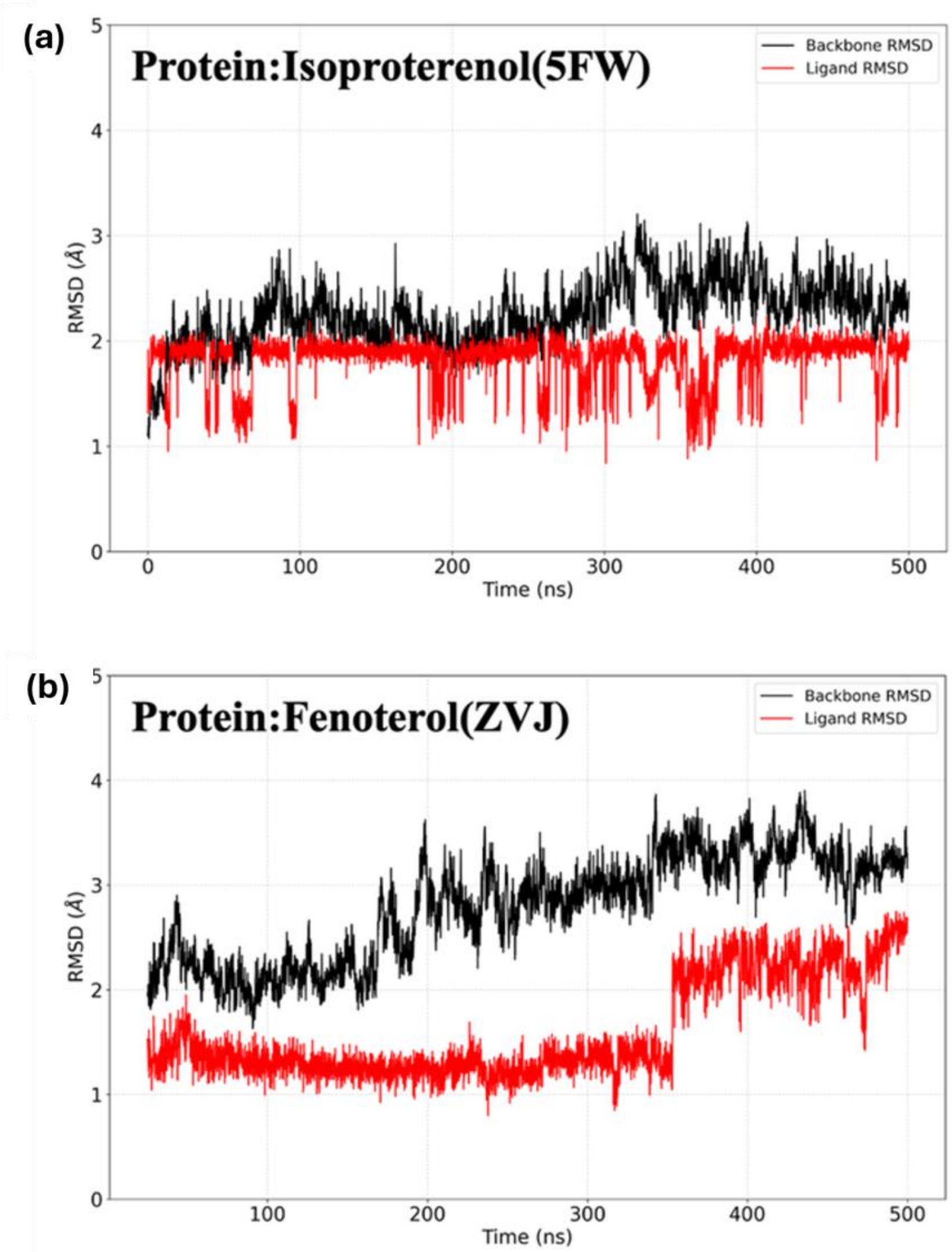


**Figure S7: RMS differences between β$_2$AR bound to ISO and FENO.** RMS Difference (RMSD) data of the β$_2$AR backbone (black) and ligand (red) non-hydrogen atoms during the MD simulations. (A) ISO-β$_2$AR complex and (B) FENO-β$_2$AR complex. RMSD was calculated relative to the initial conformations of β$_2$AR and the ligands, respectively. ISO: isoproterenol; FENO: fenoterol.

**Figure S8**

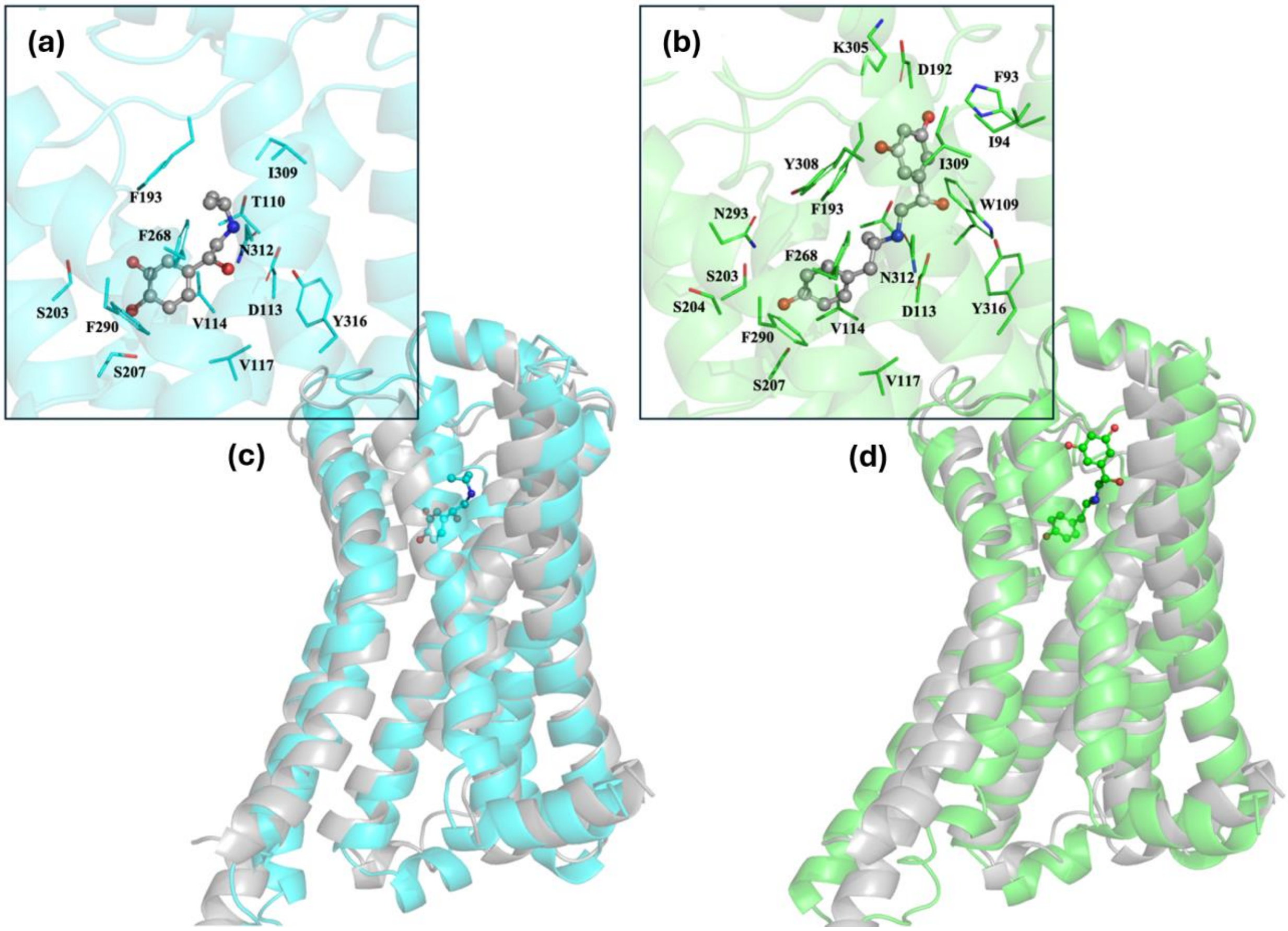


**Figure S8: β$_2$AR structural changes between ISO and FENO.** Structural comparison of the β$_2$AR conformations in the presence of the two agonists, ISO (cyan) and FENO (green), obtained from the MD simulations and the crystal structure of the ISO-β$_2$AR complex. (A-B) A detailed close-up view of the orthosteric pockets and (C-D) overall β$_2$AR structures, including the respective ligands. ISO: isoproterenol; FENO: fenoterol.

**Figure S9**

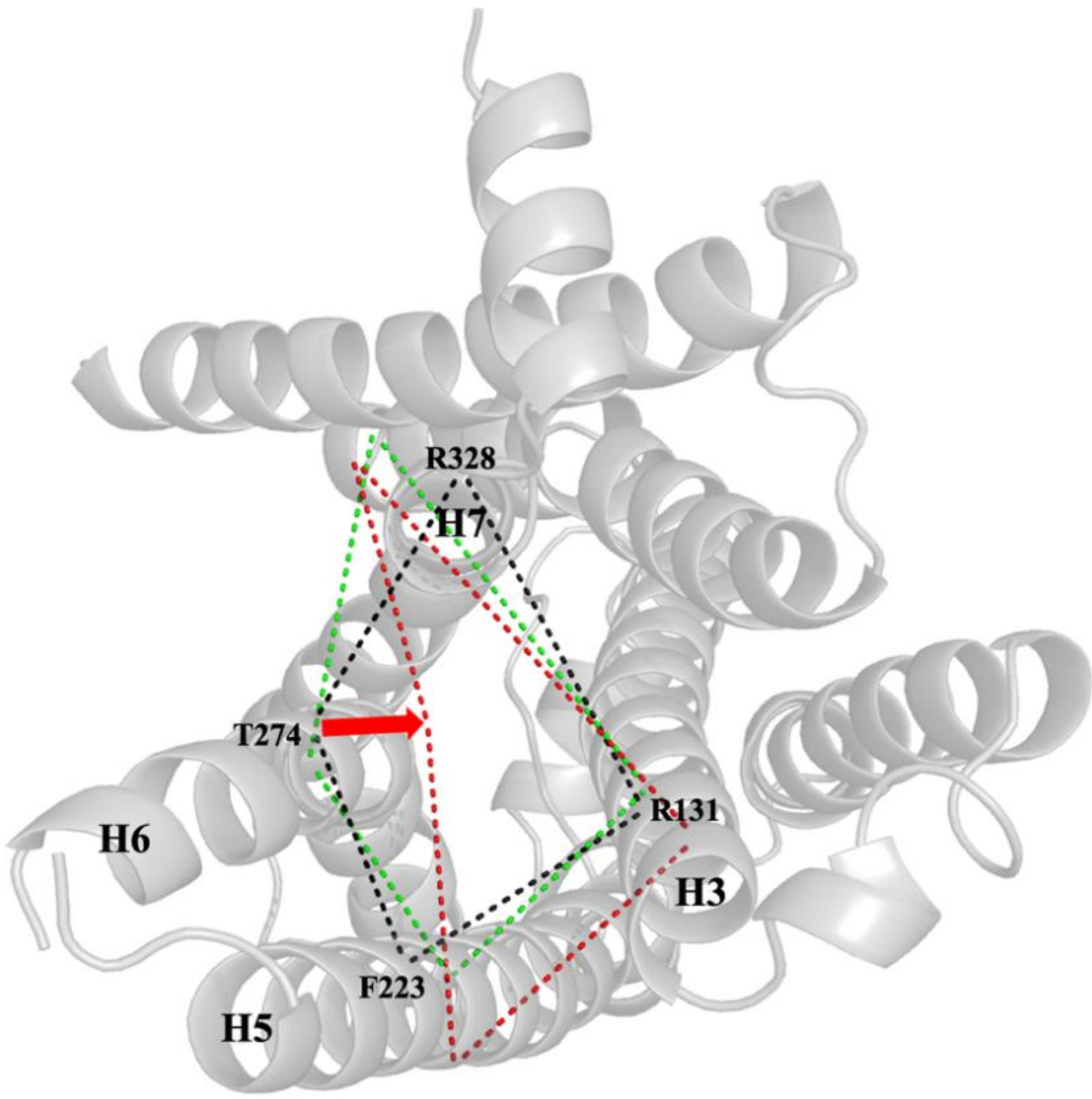


**Figure S9: Differences in transmembrane mobility between ISO- and FENO- induced $\beta_2$AR.** A bottom view of $\beta_2$AR's allosteric pocket in the presence of different orthosteric ligands. Black dotted lines indicate distances of helices in the crystal structure of the active state of the $\beta_2$AR (grey), and green and red dotted lines indicate the distances from the MD simulations in the presence of ISO (green) and FENO (red) bound to the orthosteric pocket, respectively. The red arrow indicates the shift of transmembrane helix 6 (residue Tyr274$^{6.36}$) in the presence of FENO. ISO: isoproterenol; FENO: fenoterol.

**Table S1**

(a)

## HEK293-$\beta_2$AR cAMP - Emax

| | Veh | PAM37 | NAM36 | NAM42 |
|---|---|---|---|---|
| ISO | 888.0 ± 31.9 | 1964.0 ± 41.4* | 465.4 ± 15.6* | 317.3 ± 11.7* |
| FORM | 832.6 ± 84.9 | 1159.0 ± 120.5* | 564.4 ± 65.0* | 416.6 ± 40.2* |
| ALB | 435.0 ± 14.6 | 561.9 ± 25.8* | 229.1 ± 12.2* | 210.8 ± 20.9* |
| SALM | 604.0 ± 35.2 | 939.4 ± 64.7* | 518.4 ± 9.4 | 452.7 ± 15.7 |
| TERB | 790.4 ± 30.2 | 1511.0 ± 27.9* | 814.8 ± 57.3 | 728.8 ± 30.1 |
| EPI | 1010.0 ± 15.3 | 1434.0 ± 35.7* | 792.8 ± 14.8* | 438.4 ± 5.7* |
| NorEPI | 296.2 ± 15.5 | 364.3 ± 26.6 | 225.2 ± 10.6 | 203.5 ± 12.6* |
| FENO | 707.4 ± 57.3 | 727.9 ± 72.2 | 710.9 ± 68.7 | 722.6 ± 60.3 |

(b)

## HEK293-$\beta_2$AR cAMP - $pEC_{50}$

| | Veh | PAM37 | NAM36 | NAM42 |
|---|---|---|---|---|
| ISO | 6.3 ± 0.1 | 6.3 ± 0.0 | 6.1 ± 0.1* | 6.3 ± 0.0 |
| FORM | 6.9 ± 0.3 | 6.5 ± 0.3 | 7.0 ± 0.1 | 6.2 ± 0.1 |
| ALB | 6.0 ± 0.1 | 5.9 ± 0.0 | 5.4 ± 0.0* | 5.8 ± 0.1 |
| SALM | 6.7 ± 0.3 | 6.7 ± 0.1 | 6.7 ± 0.2 | 6.1 ± 0.1 |
| TERB | 6.3 ± 0.1 | 6.3 ± 0.0 | 6.1 ± 0.1 | 6.2 ± 0.1 |
| EPI | 5.9 ± 0.1 | 5.9 ± 0.1 | 5.7 ± 0.1 | 6.3 ± 0.1* |
| NorEPI | 5.9 ± 0.0 | 5.7 ± 0.1 | 6.0 ± 0.1 | 5.8 ± 0.1 |
| FENO | 6.5 ± 0.0 | 6.6 ± 0.1 | 6.5 ± 0.0 | 6.6 ± 0.1 |

**Table S1. Emax and $EC_{50}$ for cAMP Generation in HEK293 expressing human $\beta_2$AR.** (A) Emax and (B) $EC_{50}$ calculated from dose-response curves from cells stimulated with increasing concentrations of β-agonists in the presence of vehicle or 100 nM AMs. * $p < 0.05$ β-agonist vs AMs + β-agonist.

**Table S2**

(a) HASM cAMP - Emax

| | Veh | PAM37 | NAM36 | NAM42 |
|---|---|---|---|---|
| ISO | 317.7 ± 5.0 | 520.4 ± 12.6* | 185.9 ± 5.5* | 122.1 ± 3.4* |
| FORM | 281.4 ± 25.8 | 374.6 ± 29.6* | 173.1 ± 11.7* | 167.2 ± 11.7* |
| ALB | 97.4 ± 3.3 | 126.6 ± 5.7* | 55.1 ± 1.6* | 35.9 ± 0.7* |
| SALM | 63.2 ± 5.1 | 75.5 ± 7.2 | 52.2 ± 3.4 | 48.7 ± 5.1 |
| TERB | 236.5 ± 9.6 | 496.6 ± 34.1* | 240.7 ± 10.7 | 233.5 ± 7.6 |
| EPI | 283.5 ± 8.4 | 392.6 ± 17.1* | 177.4 ± 12.0* | 162.2 ± 6.5* |
| NorEPI | 93.7 ± 3.9 | 112.4 ± 4.8* | 52.3 ± 3.2* | 46.2 ± 1.1* |
| FENO | 250.8 ± 5.3 | 242.4 ± 9.8 | 227.3 ± 12.6 | 245.3 ± 8.9 |

(b) HASM cAMP - $pEC_{50}$

| | Veh | PAM37 | NAM36 | NAM42 |
|---|---|---|---|---|
| ISO | 6.2 ± 0.1 | 6.1 ± 0.1 | 5.8 ± 0.1 | 5.9 ± 0.3 |
| FORM | 7.4 ± 0.1 | 7.2 ± 0.2 | 7.8 ± 0.2 | 8.0 ± 0.1 |
| ALB | 6.4 ± 0.0 | 6.1 ± 0.2 | 6.1 ± 0.1 | 6.6 ± 0.2 |
| SALM | 7.6 ± 0.1 | 7.9 ± 0.1 | 8.2 ± 0.1* | 7.9 ± 0.2 |
| TERB | 6.2 ± 0.1 | 6.4 ± 0.1 | 6.0 ± 0.1 | 6.1 ± 0.1 |
| EPI | 7.5 ± 0.1 | 7.9 ± 0.1 | 8.3 ± 0.0 | 2.2 ± 2.8 |
| NorEPI | 7.4 ± 0.1 | 8.0 ± 0.0* | 8.3 ± 0.0* | 8.2 ± 0.1* |
| FENO | 6.1 ± 0.1 | 6.2 ± 0.0 | 6.1 ± 0.1 | 6.0 ± 0.0 |

**Table S2. Emax and $EC_{50}$ for cAMP Generation in primary human ASM cells.** (A) Emax and (B) $EC_{50}$ calculated from dose-response curves from cells stimulated with increasing concentrations of β-agonists in the presence of vehicle or 100 nM AMs. *$P < 0.05$ β-agonist vs AMs + β-agonist.

**Table S3**

(a) HEK βArrestin$_2$-Recruitment - Emax

| | Veh | PAM37 | NAM36 | NAM42 |
|---|---|---|---|---|
| ISO | 109.6 ± 6.4 | 103.2 ± 5.9 | 103.1 ± 6.0 | 101.2 ± 3.1 |
| FORM | 83.1 ± 2.8 | 83.5 ± 2.7 | 83.3 ± 2.8 | 84.2 ± 1.5 |
| ALB | 52.5 ± 10.0 | 49.6 ± 9.1 | 50.4 ± 9.3 | 50.2 ± 4.8 |
| SALM | 12.9 ± 5.0 | 10.2 ± 2.1 | 11.0 ± 3.4 | 11.7 ± 2.1 |
| TERB | 64.3 ± 1.3 | 65.8 ± 1.7 | 67.9 ± 1.6 | 68.9 ± 0.7 |
| EPI | 102.0 ± 3.2 | 100.1 ± 1.9 | 105.4 ± 2.5 | 107.5 ± 1.4 |
| NorEPI | 84.2 ± 3.6 | 83.7 ± 2.6 | 83.4 ± 2.3 | 83.5 ± 1.4 |
| FENO | 70.3 ± 1.7 | 67.9 ± 1.4 | 65.8 ± 1.4 | 68.4 ± 0.9 |

(b) HEK βArrestin$_2$-Recruitment - pEC$_{50}$

| | Veh | PAM37 | NAM36 | NAM42 |
|---|---|---|---|---|
| ISO | 8.2 ± 0.1 | 8.3 ± 0.1 | 8.3 ± 0.1 | 8.3 ± 0.1 |
| FORM | 6.7 ± 0.1 | 6.7 ± 0.1 | 6.7 ± 0.1 | 6.7 ± 0.0 |
| ALB | 5.5 ± 0.3 | 5.5 ± 0.3 | 5.5 ± 0.3 | 5.5 ± 0.2 |
| SALM | 6.1 ± 1.0 | 6.8 ± 0.5 | 6.7 ± 0.8 | 6.5 ± 0.5 |
| TERB | 6.9 ± 0.0 | 6.9 ± 0.1 | 6.9 ± 0.1 | 6.9 ± 0.0 |
| EPI | 7.8 ± 0.1 | 7.8 ± 0.1 | 7.7 ± 0.1 | 7.7 ± 0.0 |
| NorEPI | 5.8 ± 0.1 | 5.7 ± 0.0 | 5.8 ± 0.0 | 5.7 ± 0.0 |
| FENO | 7.0 ± 0.1 | 7.0 ± 0.0 | 7.0 ± 0.0 | 7.0 ± 0.0 |

**Table S3. Emax and EC$_{50}$ for β-arrestin recruitment to the β$_2$AR.** (A) Emax and (B) EC$_{50}$ calculated from dose-response curves from cells stimulated with increasing concentrations of β-agonists in the presence of vehicle or 100 nM AMs.

**Table S4**

(a) **Bronchodilation mPCLS - Emax**

| | Veh | PAM37 | NAM36 | NAM42 |
|---|---|---|---|---|
| ISO | 24.5 ± 1.3 | 41.8 ± 2.2* | 16.8 ± 2.1* | 12.3 ± 1.1* |
| FORM | 17.3 ± 2.0 | 27.7 ± 1.5* | 8.7 ± 0.9* | 3.3 ± 0.6* |
| ALB | 11.3 ± 0.9 | 18.7 ± 2.0* | 8.0 ± 0.6 | 4.0 ± 1.0* |
| SALM | 12.0 ± 0.6 | 23.0 ± 1.2* | 11.7 ± 0.9 | 4.7 ± 0.9* |
| TERB | 22.0 ± 1.2 | 33.3 ± 1.3* | 13.5 ± 0.6* | 11.5 ± 0.5* |
| EPI | 20.8 ± 1.5 | 37.5 ± 1.7* | 11.3 ± 1.5 | 9.3 ± 0.9* |
| FENO | 26.3 ± 2.2 | 28.0 ± 2.1 | 28.0 ± 1.4 | 27.8 ± 1.6 |

(b) **Bronchodilation mPCLS – p$EC_{50}$**

| | Veh | PAM37 | NAM36 | NAM42 |
|---|---|---|---|---|
| ISO | 7.1 ± 0.1 | 7.1 ± 0.1 | 5.9 ± 0.3 | 4.5 ± 0.9 |
| FORM | 6.2 ± 0.1 | 6.4 ± 0.1 | 6.7 ± 0.4 | 5.7 ± 0.5 |
| ALB | 5.3 ± 0.2 | 5.5 ± 0.5 | 5.7 ± 0.7 | 5.8 ± 0.4 |
| SALM | 4.9 ± 0.2 | 6.1 ± 0.2* | 5.0 ± 0.1 | 5.7 ± 0.2 |
| TERB | 6.1 ± 0.2 | 6.1 ± 0.1 | 6.2 ± 0.2 | 6.1 ± 0.0 |
| EPI | 6.4 ± 0.1 | 6.7 ± 0.1 | 6.2 ± 0.0 | 6.0 ± 0.1 |
| FENO | 6.1 ± 0.1 | 6.0 ± 0.0 | 6.1 ± 0.1 | 6.1 ± 0.1 |

**Table S4. Emax and $EC_{50}$ for bronchodilation of murine PCLS.** (A) Emax and (B) $EC_{50}$ calculated from dose-response curves from murine lung slices stimulated with increasing concentrations of β-agonists in the presence of vehicle or 100 nM AMs. *$P < 0.05$ β-agonist vs AMs + β-agonist.

**Table S5**

(a) HASM cAMP - Emax

| | Veh | PAM37 | NAM36 | NAM42 |
|---|---|---|---|---|
| ISO | 0% | 64% | -41% | -62% |
| FORM | 0% | 33% | -38% | -41% |
| ALB | 0% | 30% | -43% | -63% |
| SALM | 0% | 19% | -17% | -23% |
| TERB | 0% | 110% | 2% | -1% |
| EPI | 0% | 38% | -37% | -43% |
| NorEPI | 0% | 20% | -44% | -51% |
| FENO | 0% | -3% | -9% | -2% |

(b) HEK293-$\beta_2$AR cAMP - Emax

| | Veh | PAM37 | NAM36 | NAM42 |
|---|---|---|---|---|
| ISO | 0% | 121% | -48% | -64% |
| FORM | 0% | 39% | -32% | -50% |
| ALB | 0% | 29% | -47% | -52% |
| SALM | 0% | 56% | -14% | -25% |
| TERB | 0% | 91% | 3% | -8% |
| EPI | 0% | 42% | -22% | -57% |
| NorEPI | 0% | 23% | -24% | -31% |
| FENO | 0% | 3% | 0% | 2% |

(c) mPCLS - Emax

| | Veh | PAM37 | NAM36 | NAM42 |
|---|---|---|---|---|
| ISO | 0% | 71% | -31% | -50% |
| FORM | 0% | 60% | -50% | -81% |
| ALB | 0% | 65% | -29% | -65% |
| SALM | 0% | 92% | 6% | -61% |
| TERB | 0% | 51% | -39% | -48% |
| EPI | 0% | 92% | 6% | -61% |
| FENO | 0% | 6% | 6% | 6% |

(d) Gαs-Recruitment - Emax

| | Veh | PAM37 | NAM36 | NAM42 |
|---|---|---|---|---|
| ISO | 0% | 84% | -35% | -64% |
| FORM | 0% | 99% | -23% | -49% |
| ALB | 0% | 34% | -30% | -59% |
| SALM | 0% | 52% | -25% | -46% |
| TERB | 0% | 57% | -6% | -33% |
| EPI | 0% | 97% | -25% | -46% |
| NorEPI | 0% | 20% | -19% | -37% |
| FENO | 0% | -1% | 2% | -1% |

**Table S5. Magnitude increases in Emax.** Calculated Emax values from cAMP generation in (A) human ASM cells, (B) HEK293 cells transfected with human $\beta_2$AR, (C) Gαs-recruitement, and (D) bronchodilation of murine PCLS were normalized to their respective β-agonist. The values shown represent are the percent increase/decrease relative to the β-agonist-treated condition.

**Table S6**

(a) Ionic Lock Distances

| Complex | Ionic lock distance (Å) |
|---|---|
| $\beta_2$AR:ISO (5FW) | 16.4 ± 0.62 |
| $\beta_2$AR:FENO (ZVJ) | 12.3 ± 0.38 |

(b) Cα Distances

| | Crystal data (PDB ID: 3SN6) | $\beta_2$AR:ISO (5FW) | $\beta_2$AR:FENO (ZVJ) |
|---|---|---|---|
| R131-F223 | 10.9 | 11.15 ± 0.32 | 12.71 ± 0.45 |
| F223-T274 | 10.2 | 10.49 ± 0.14 | 13.29 ± 0.22 |
| T274-R328 | 11.5 | 13.36 ± 0.57 | 11.85 ± 0.36 |
| R328-R131 | 15.0 | 16.82 ± 0.64 | 19.87 ± 0.69 |

f

**Table S6. Ionic Lock and Cα atom distances between ISO and FENO.** (A) Ionic lock distance (Å) between Cα of Arg131$^{3.50}$ to Cα of Glu268$^{6.30}$ collected from the last 100ns of three trials. (B) Cα atom distances (Å) between Arg131$^{3.50}$ (TM3), Phe223$^{5.62}$ (TM5), Tyr274$^{6.36}$ (TM6), and Arg328$^{7.55}$ (TM7) collected from the last 100ns of three trials. Values represent mean ± standard deviation. ISO: isoproterenol; FENO: fenoterol.
